# Ionic Conductivity of Nanocrystalline and Amorphous Li_10_GeP_2_S_12_: The Detrimental Impact of Local Disorder on Ion Transport

**DOI:** 10.1021/jacs.1c13477

**Published:** 2022-05-24

**Authors:** Lukas Schweiger, Katharina Hogrefe, Bernhard Gadermaier, Jennifer L. M. Rupp, H. Martin R. Wilkening

**Affiliations:** † Institute of Chemistry and Technology of Materials, Christian Doppler Laboratory for Lithium Batteries, Graz University of Technology (NAWI Graz), Graz 8010, Austria; ‡ Electrochemical Materials, Department of Materials Science and Engineering, Massachusetts Institute of Technology, Cambridge, Massachusetts 02139, United States; § Electrochemical Materials, Department of Electrical Engineering & Computer Science, Massachusetts Institute of Technology, Cambridge, Massachusetts 02139, United States

## Abstract

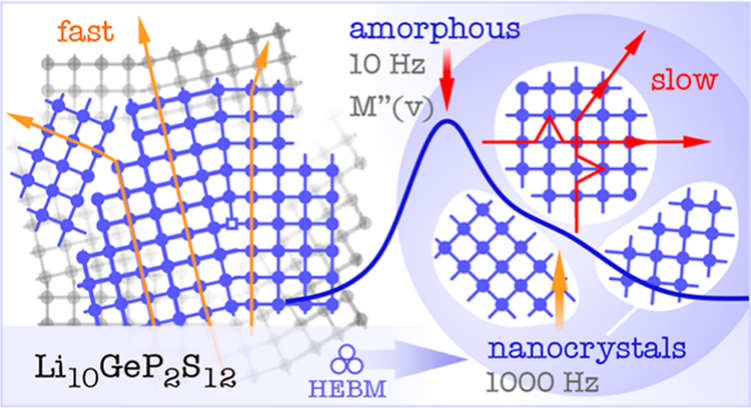

Solids with extraordinarily high Li^+^ dynamics are key for high performance all-solid-state batteries. The thiophosphate Li_10_GeP_2_S_12_ (LGPS) belongs to the best Li-ion conductors with an ionic conductivity exceeding 10 mS cm^–1^ at ambient temperature. Recent molecular dynamics simulations performed by Dawson and Islam predict that the ionic conductivity of LGPS can be further enhanced by a factor of 3 if local disorder is introduced. As yet, no experimental evidence exists supporting this fascinating prediction. Here, we synthesized nanocrystalline LGPS by high-energy ball-milling and probed the Li^+^ ion transport parameters. Broadband conductivity spectroscopy in combination with electric modulus measurements allowed us to precisely follow the changes in Li^+^ dynamics. Surprisingly and against the behavior of other electrolytes, bulk ionic conductivity turned out to decrease with increasing milling time, finally leading to a reduction of σ_20°C_ by a factor of 10. ^31^P, ^6^Li NMR, and X-ray diffraction showed that ball-milling forms a structurally heterogeneous sample with nm-sized LGPS crystallites and amorphous material. At −135 °C, electrical relaxation in the amorphous regions is by 2 to 3 orders of magnitude slower. Careful separation of the amorphous and (nano)crystalline contributions to overall ion transport revealed that in both regions, Li^+^ ion dynamics is slowed down compared to untreated LGPS. Hence, introducing defects into the LGPS bulk structure *via* ball-milling has a negative impact on ionic transport. We postulate that such a kind of structural disorder is detrimental to fast ion transport in materials whose transport properties rely on crystallographically well-defined diffusion pathways.

## 1 Introduction

Li^+^ ion batteries are the workhorses for energy storage and play a vital role in most consumer electronics. Their usage will become even more abundant with the continuing electrification of transportation and their introduction to large-scale grid storage.^1−4^ Unfortunately, conventional Li^+^ ion batteries start approaching their energy density limits.^5,6^ Although for many applications the problem of thermal runaway and flammability has almost been overcome, increased safety concerns are discussed for larger cells used in electric vehicles or for stationary grid storage.^3,7−9^ One concept to overcome these current limitations is the replacement of the liquid organic electrolyte by a solid (crystalline) electrolyte.^3,5,10^ Although solid electrolytes and so-called all-solid-state batteries hold the promise of being superior to conventional Li-ion batteries, many hurdles need to be surmounted. These are especially related to insufficient ionic conductivity and insufficient electrochemical stability.^11^ The latter is tightly connected to preventing the undesired processes occurring near or at the electrode–electrolyte interfaces.^12,13^

**Figure 1:**
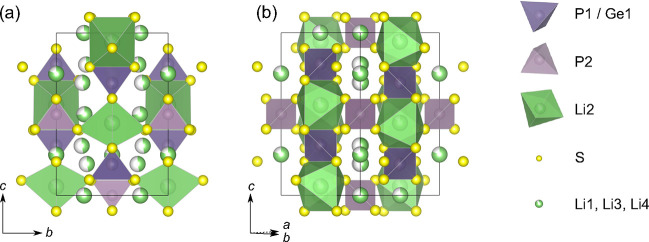
Tetrahedral unit cell of Li_10_GeP_2_S_12_ with the space group *P*4_2_/*nmc* (no. 137) depicted from different perspectives showing (a) channel of Li^+^ ions along the *c*-direction and (b) chain of edge-sharing LiS_6_–(Ge/P)S_4_ polyhedra. The phosphorous ions occupy the 4d (P1/Ge1, dark violet) and 2b (P2, light violet) sites, and the former site is shared between the phosphorous and the germanium ions in a 1:1 ratio. The sulfur resides on 8g sites and tetrahedrally coordinates the P^5+^ and Ge^4+^ ions. The lithium occupies four sites, namely, 16h (Li1), 4d (Li2), 8f (Li3), and 4c (Li4), each with an occupation factor <1. The exact distributions and site symmetries of Li^+^ ions in LGPS are still not completely resolved. Together with the octahedrally coordinated Li2 site (4d), the [P1S_4_]^3–^ and [Ge1S_4_]^4–^ (2b) tetrahedra constitute chains along the *c*-direction of the unit cell. These chains are linked by the [P2S_4_]^3–^ groups. The corresponding polyhedra are shown in the figure. Li2 is often regarded as “inactive” in the conduction process, and therefore, it constitutes, together with the other polyanion groups, the structural framework. The diffusion pathways of the Li^+^ ions can be seen along the *c*-direction (Li1 and Li3) and in the *ab* plane (Li1 and Li4) of the crystal structure. Li2 might, however, also take part in the ion migration process.

Some of the most promising candidates to act as powerful ceramic electrolytes include oxides such as perovskite-type,^14^ NASICON-type,^15^ LISICON-type^16,17^ and garnet-type materials,^18^ phosphates,^19^ and thio-phosphates such as the thio-LISICONS,^20,21^ Li-argyrodites,^22^ LTPS,^23^and Li_10_GeP_2_S_12_ (LGPS) as well as its relatives;^24^ see Kim *et al.*(25) LGPS, for instance, shows remarkably high ionic conductivity values as high as 12 mS cm^–1^ at room temperature.^24^ Its crystal structure, the foundation for the exceptional high ionic conductivity, is illustrated in Figure 1.

Balancing the amount and nature of amorphous and crystalline phases inside these materials is a crucial factor influencing the ionic conductivity. While some thiophosphate systems show higher conductivity in an amorphous form, other systems depend on high crystallinity.^26−28^ Although local structures of Li have been characterized by many workgroups,^29−33^ the exact arrangement and occupation of the Li^+^ sites and the origin of the change in activation energy with increasing temperature still remain a matter of debate.^34^ The latter refers to the decrease in activation energy *E*_a_ of ionic transport at sub-ambient temperature, that is, at approximately −20 °C, from about 0.31 eV (low temperature regime) to 0.17 eV (high temperature regime).^24,34^ In the literature, different explanations are provided for this phenomenon such as the involvement of blocking grain boundaries (g.b.), which might govern ion transport at lower temperatures,^31^ and the presence of a diffuse phase transition.^35^ Other possible explanations include (i) changes in the rate-limiting diffusion step as well as (ii) the transition from quasi one-dimensional to three-dimensional transport at elevated temperatures.^33−35^

Li_10_GeP_2_S_12_ is the prototype of a larger set of materials with similar structures.^34^ They had been prepared in the hope that even higher ionic conductivities can be reached. Examples include Li_10_SnP_2_S_12_ (4 mS cm^–1^),^36^ Li_11_Si_2_PS_12_ (4 mS cm^–1^),^37^ and Li_9.54_Si_1.74_P_1.44_S_11.7_Cl_0.3_ (25 mS cm^–1^),^38^ in particular. A selection of the as yet synthesized LGPS-type materials is listed in Table S1. Many of the derivatives show conductivity values very similar to LGPS, revealing that a threshold in ionic conductivity of this class of materials might have been reached. Also, a recent text mining study on the synthesis conditions for LGPS of over 900 papers comes with high statistics to a similar conclusion.^39^ Most of the studies focus on the crystal chemistry of LGPS-type materials, that is, they investigate substitution effects in ideal solid solutions on overall ion conductivity by introducing iso- or aliovalent ions. It is known within the wider field of functional ceramics, that high-entropy and doped solid-state ionic conductors, in particular, reveal often inhomogeneous distributions of dopant cations over grain boundaries leading to space charge zones and local 2nd order phase deteriorations. The smaller the average grain size, that is, the higher the grain boundary over the grain volume, the more such effects come into play. Importantly, very recent findings on Li^+^ conducting oxides even reveal that this behavior can lead to substantial alterations of transference numbers and fluctuations of the reduction of Li^+^ near the grain boundaries,^40,41^ which can affect dendrite formation. In that light, it is surprising that till date engineering the conduction properties of Li_10_GeP_2_S_12_ by altering the crystallite size has not been a substantial matter of experimental study yet.

Based on their computational results, Dawson and Islam proposed^42^ that the already high ionic conductivity of LGPS might be further increased by a factor of 3 when reducing the crystallite size from the conventional micrometer range down to a grain volume of 10 nm^3^.^42^ They explained the slight enhancement seen through changes of the local Li^+^ ion coordination, that is, local disorder. These structural changes are assumed to facilitate the slower diffusion process in the *ab*-plane of LGPS. As a result, in nanosized LGPS, the diffusion pathways are more isotropic than in the bulk material. According to the simulations, the ionic transport in LGPS shifts from a preferential diffusion along the *c*-direction to quasi 3D for the nanosized structure. Additionally, it is reported that for a structure made of grains with a volume of 10 nm^3^ the diffusion length for Li-ion transport is significantly reduced, which results in increased intergranular diffusion.^42^

A relatively simple and established way to decrease the average crystal size and to introduce structural (point) disorder is given by high-energy ball-milling, which is a top-down approach to prepare nm-sized crystallites.^43,44^ If starting with rather poorly conducting coarse-grained materials, many studies reported on enhanced ionic conductivities seen for the nanocrystalline single phase counterparts such as γ-LiAlO_2_,^45^ β-spodumene LiAlSi_2_O_6_,^46,47^ the glass former Li_2_B_4_O_7_,^48^ Li_2_TiO_3_,^49^ LiTaO_3_,^50^ LiNbO_3_,^51^ Li_2_S,^52^ and also thiophosphates such as argyrodite-type Li_6_PS_5_I.^53^ On the other hand, mechanically induced structural relaxation is reported to decrease the ionic conductivity for glasses that were prepared by quenching.^46,47,54,55^

For LGPS, an investigation on the structural and dynamic changes caused by treating a sample with μm-sized crystallites is still missing. The changes expected might be more important than anticipated as Li_10_GeP_2_S_12_ is a relatively soft material. Mechanical properties of LGPS are characterized by 1/3 to 1/4th of the Young’s modulus reported for oxides.^25,33,56,57^ Therefore, the current study is aimed at answering the questions: (i) to which extent ball-milling affects local structures in LGPS and (ii) whether it is able to considerably enhance ion dynamics in LGPS, as suggested theoretically.

Here, by using relatively mild milling conditions,^53^ we prepared a series of nanocrystalline LGPS samples reaching a mean crystallite diameter of 10 nm. The effect of ball-milling on LGPS turned out to be twofold. Indeed, we were able to prepare nanocrystalline LGPS, but X-ray powder diffraction and high-resolution ^31^P NMR showed that even under these conditions crystalline LGPS partly transforms into an amorphous material. At longer milling times an almost fully amorphous sample is obtained. Most likely, the samples are to be regarded as nm-sized crystallites of LGPS embedded in an amorphous matrix. This morphology might not only reveal rapid ion dynamics because of the nm-sized LGPS regions but could also provide a percolating network of fast transport pathways along the amorphous–crystalline interfacial regions generated. Such phenomena, which take advantage of space charge effects, have been reported for LiF films on SiO_2_,^58^ LiF/TiO_2_ systems,^59^ glass ceramic LiAlSiO_4_,^60^ or LiBH_4_/Al_2_O_3_.^61^ Additionally, amorphous and/or strained LGPS promises better chemical stability,^62^ giving its preparation and characterization importance for future cell applications.

In the present case, we do, however, observe that any kind of structural disorder introduced into LGPS, be it extended amorphous regions or defects in the bulk structure, slows down macroscopic ion transport. This finding also seems to hold good for the interfacial regions.^63^ Therefore, we conclude that for materials with crystallographically well-defined pathways guaranteeing rapid Li^+^ transport, as it is the case for LGPS, defects and site disorder deteriorate or even interrupt the lanes for rapid Li^+^ exchange. In the style of Shakespeare’s principle *order versus disorder*, our results emphasize the importance of controlling structural (site) disorder and defect chemistry to ensure fast ion transport in LGPS-type electrolytes.

## 2 Experimental Section

We synthesized Li_10_GeP_2_S_12_ by following a classical solid-state preparation route. Stoichiometric amounts of the starting materials, Li_2_S (Alfa Aesar 99.9%), P_2_S_5_ (Sigma-Aldrich 99%), and GeS_2_ (abcr 99.99%), were weighed in and put into a ZrO_2_ milling vial (45 mL) together with 180 ZrO_2_ balls (5 mm in diameter, the ball-to-powder ratio was approximately 20:1). A planetary ball mill (Fritsch Pulverisette 7 Premium line) was employed to treat the mixture mechanically. The powder was treated for 40 h at 380 rounds per min (rpm) with alternating cycles of 15 min milling and 15 min pause to avoid extensive heating, summing up to a net milling time of 20 h. Pellets were pressed and sealed in an evacuated quartz tube. The sealed samples were annealed at 550 °C (1 °C min^–1^) for 8 h. The annealed pellets were ground using a mortar and pestle. To nanostructure the as-synthesized LGPS powders, they were milled together with 60 ZrO_2_ balls (5 mm, the ball-to-powder ratio was approximately 30:1) for different milling times; see Table S4. All steps were performed in an Ar-filled glovebox with the H_2_O and O_2_ levels both being lower than 0.1 ppm.

**Figure 2:**
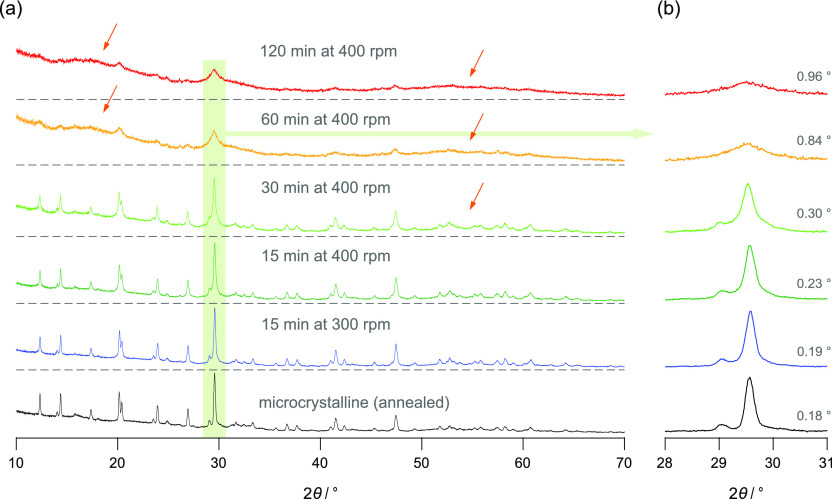
(a) Stacked plot of the X-ray diffraction patterns of the as-synthesized microcrystalline sample (shown at the bottom) and the nanocrystalline LGPS samples prepared by milling for the durations and at the rotational speeds indicated. The increased background signal at low diffraction angles originates from both instrumental sources and the Kapton foil used to protect the sample from any reaction with air. Despite this feature, broader humps emerge upon milling, which are indicated by arrows. Additionally, the reflections broaden owing to size effects and strain introduced. Importantly, no other phases than LGPS are formed during the milling procedures. A largely amorphous sample is obtained after 120 min of milling. (b) Magnification of the main reflection of LGPS [miller indices (203)] located at approximately 29.5° to illustrate X-ray peak broadening. Numbers refer to the widths (full width at half maximum) of this reflection deduced from appropriate pseudo-Voigt functions used to approximate the shape of the signals.

Powder X-ray diffraction (PXRD) patterns were recorded either using a Rigaku MiniFlex (Bragg Brentano geometry, Cu K_α_ radiation) or using a Rigaku SmartLab (capillaries, Cu K_α_ radiation). During the measurements, the samples were protected from any reaction with traces of moisture either by using an air-sensitive sample holder (MiniFlex) or by using glass capillaries that were sealed with grease and parafilm (SmartLab device). The exact experimental parameters differ from instrument to instrument and are also provided in the Supporting Information; see Figures 2 and S1.

To carry out impedance measurements, we pelletized the powders and applied Au electrodes (50–100 nm) on top of that by using a Leica sputter coater. Complex impedances were measured with a Novocontrol concept 80 spectrometer over a broad frequency range covering several orders of magnitude, that is, from 10^–2^ Hz to 10 MHz. Conductivity isotherms and Nyquist plots were recorded under a nitrogen atmosphere and as a function of temperature *T*. Measurements were performed from 138 to 373 K in steps of 20 K. A Quatro Cryosystem (Novocontrol) was employed to control and to monitor the temperature in the sample chamber. For this purpose, a stream of freshly evaporated nitrogen gas, passing a heating unit, was used to adjust the temperature in the chamber. Mounting the sample in the cell of the Novocontrol spectrometer was carried out as quickly as possible to minimize exposure to air. Before each temperature run, we equilibrated the sample at 373 K for at least 5 min. Then, we started to record the heating and cooling runs.

For Raman spectroscopy, the powder sample was thoroughly ground and filled into a glass capillary, which was then sealed using grease and parafilm. The measurements were carried out using a Thermo Scientific DXR 2 Raman microscope operating with a 532 nm laser source; see the Supporting Information for further details.

High-resolution ^31^P and ^6^Li (magic angle spinning) MAS NMR spectra were acquired on a Bruker Avance III 500 MHz spectrometer using 2.5 mm ZrO_2_ rotors that were rotated at a speed of ν_rot_ = 25 kHz at ambient bearing gas conditions. The spectra were referenced to CaHPO_4_ (Fluka, >97%) and to CH_3_COOLi·2H_2_O (Sigma-Aldrich, ≥97%), assigning chemical shifts of −1.5 ppm (upfield signal) and 0 ppm, respectively. For each measurement, the pulse lengths, the number of scans, recycle delays, reference phases, and exact resonance frequencies were carefully adjusted to obtain an optimal free induction decay (FID) under on-resonance conditions; see also Table S2 and Figure S7. The FIDs were Fourier transformed without any further manipulation procedures to yield the ^7^Li and ^31^P MAS NMR spectra shown here.

## 3 Results and Discussion

### 3.1 X-ray Diffraction and MAS NMR

**Figure 3:**
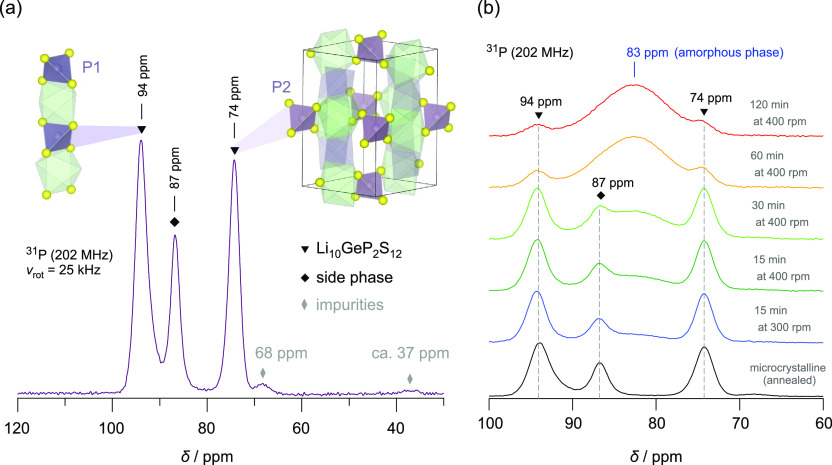
(a) ^31^P MAS NMR spectrum (202.5 MHz, rotational frequency 25 kHz) of microcrystalline Li_10_GeP_2_S_12_. The chemical shifts are referenced to ^31^P signal in CaHPO_4_. Lines at isotropic chemical shifts of 94 and 74 ppm are of equal intensity. They are attributed to the 4d (P1) and 2b (P2) sites in the LGPS structure, respectively. The signal at 87 ppm is assigned to the side phase formed already during synthesis of LGPS. Lines with very low intensity, which appear at 68 and 37 ppm, respectively, indicate oxygen-containing [PS_4–*n*_O_*n*_]^3–^ units; see text for further details. (b) ^31^P MAS NMR spectra of microcrystalline LGPS and ball-milled LGPS obtained after the milling times listed. Upon milling, a broad signal emerges (83 ppm) that dominates the ^31^P NMR response of the sample treated for 120 min. See text for further discussion.

The impact of high-energy ball-milling on overall structure and morphology of the as-synthesized microcrystalline LGPS was probed by X-ray powder diffraction and ^31^P MAS NMR. In Figure 2a,b, the powder pattern of the starting material, that is, unmilled LGPS is shown. As revealed by Rietveld refinement (see Figures S1 and S2) and supported by Raman spectroscopy (see Figure S3), LGPS was successfully synthesized^30^ with a minor side phase. This phase is also seen in ^31^P MAS NMR (Figure 3). It seems to be a side phase almost universally present in LGPS samples prepared by the solid-state reaction.^37,64^ While most authors attribute this phase to orthorhombic β-Li_3_PS_4_, some groups also considered the formation of an orthorhombic LGPS-phase.^31,65^ Due to structural similarity of the three structures and the identical chemical environment of P, a clear identification of the crystalline side phase turned out to be rather challenging; see below and the Supporting Information for further discussion.

Here, the broadening of the XRD reflections, see especially the signal at 29.5° (Figure 2b), is ascribed to the formation of nm-sized crystallites and strain introduced during mechanical treatment. According to the Scherrer equation^66^ and considering the quality of X-ray patterns of nanocrystalline materials, we estimated that the mean crystallite diameter takes a value of only 10 nm after the sample has been treated for 120 min (400 rpm), as outlined in the Supporting Information.

Furthermore, broader humps emerge upon milling (see arrows in Figure 2a), which we assign to the formation of structurally amorphous regions. Hence, we conclude that for the samples milled for longer times (≥30 min), the nanosized LGPS crystallites are embedded in an amorphous matrix or at least covered by an amorphous layer. The indication of amorphous regions by X-ray diffraction is fully underpinned by ^31^P MAS NMR, and the corresponding single pulse NMR spectra are shown in Figure 3. Judging from the chemical shifts of both the amorphous and the crystalline phase, which do not shift upon milling, we assume that disordered LGPS is characterized by the same stoichiometry as ordered LGPS; see also below. As shown by recent studies,^67^ changes in the Ge content would, for example, sensitively affect the ^31^P NMR chemical shift, which is not observed here. Therefore, we exclude any enrichment or depletion of Ge in either of the two phases.

As expected for microcrystalline LGPS, ^31^P MAS NMR reveals two distinct lines at chemical shifts δ of 94 and 74 ppm, respectively (Figure 3a). These lines, being almost equal in intensity and area, are distinctive for the P1 (4d) and P2 (2b) phosphorus sites in the LGPS structure.^31,37^ Importantly, apart from the formation of amorphous regions, no mechanochemical transformations of LGPS were induced during milling, as can be seen from the invariant ^31^P chemical shifts of LGPS, and the fact that the 1:1-ratio of the two P signals, which are diagnostic for crystalline or nanocrystalline LGPS, does not change.

The signal at 87 ppm could be attributed to the [PS_4_]^3–^ units in orthorhombic Li_3_PS_4_,^31,68^ a common concomitant of classically synthesized Li_10_GeP_2_S_12_.^29,31^ As suggested above, it also possibly mirrors orthorhombic LGPS forming the side phase. The area under this NMR signal amounts to approximately 16 to at most 20% (see the Supporting Information, Table S4).

The NMR lines seen at 68 ppm and *ca.* 37 ppm match with the chemical shifts reported for oxygen-containing [PS_4–*n*_O_*n*_]^3–^ units in oxysulfide glasses,^69,70^ and thus reveal minor contaminations. As an example, the NMR line of [PS_2_O_2_]^3–^ is reported to appear at 65 ppm, the one belonging to [PO_3_S]^3–^ is expected to be located at a chemical shift of 34 ppm. NMR lines representing S-free phosphate units [PO_4_]^3–^ (8 ppm)^69,71^ cannot be detected in our study. Note that similar lines and chemical shifts have also been reported for Li_10_SiP_2_S_12–*x*_O_*x*_ being a variant of the LGPS structure.^70^ Most probably, these oxygen-containing units originate from impurities in the starting materials or stem from traces of oxygen entering the milling beakers during mechanical treatment.

As the detailed analysis of the ^31^P MAS NMR spectrum of the as-synthesized LGPS yielded valuable insights into compositions and local structures, ^31^P NMR was also the method of choice to collect structural information on the milled samples; see Figure 3b. Upon mechanical treatment, a new and broad NMR line appears at 83 ppm; see Figure 3b. By increasing both milling time and milling speed, this new signal progressively gains in intensity until it dominates the NMR response (milling times: 60 and 120 min). We recognize that at a constant rotational speed of 400 rpm, the largest change occurs when the milling time is increased from 30 to 60 min. This observation excellently agrees with the crystallographic change of the two corresponding XRD patterns; see above. Most likely, the material transforms from a structurally disordered/distorted nanocrystalline one into a form that is predominantly amorphous.

In the literature, a ^31^P NMR line at 83 ppm was reported to reflect chain units being analogous to the metaphosphate groups in Li_2_S–P_2_S_5_ glasses.^68^ Broad NMR lines reveal disordered and distorted chemical environments that the ^31^P spins sense in nanocrystalline LGPS. Note that the chemical shift of 83 ppm reflects almost the average chemical shift value of the two ^31^P NMR lines belonging to crystalline Li_10_GeP_2_S_12_. We attribute the new line to ^31^P spins in a structurally amorphous phase that is continuously formed during ball-milling. Interestingly, the ^31^P NMR line of the side phase, possibly orthorhombic LGPS, decreases in intensity with increasing milling time. At sufficiently long milling time, it cannot be detected any longer (see Figure 3b) and is assumed to merge with that of amorphous LGPS.

To estimate the area fractions under the distinct ^31^P MAS NMR lines, we evaluated the whole ^31^P NMR response with Voigt functions; see Figure S4 and Table S3. As a result, for the sample milled for 120 min and at 400 rpm, the amount of amorphous LGPS turned out to be approximately 91 wt %. The other phase fractions of the other samples are listed in Table S4. Although such amorphization is expected after high-energy ball-milling,^72^ the current amount formed after 120 min is rather large as compared to that in other materials. After mechanical milling at similar conditions, the Li-bearing argyrodite-type Li_6_PS_5_I, for example, do contain approximately 15% of the amorphous material.^53^ Certainly, such numbers depend on the milling conditions. Nevertheless, even under harsh conditions, oxides^72^ and fluorides^73^ tend to show much lower if not marginal amounts of amorphous fractions.

**Figure 4:**
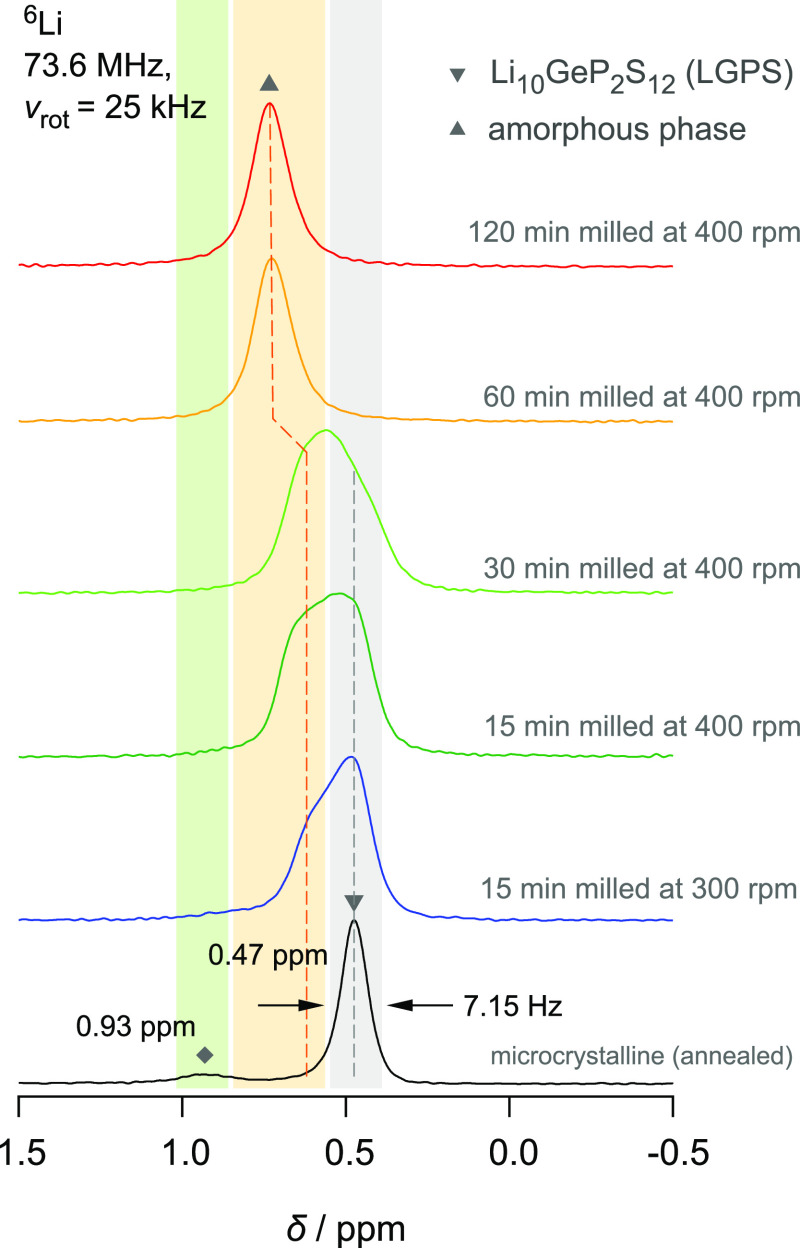
^6^Li MAS NMR spectra (73.6 MHz, 25 kHz) of microcrystalline Li_10_GeP_2_S_12_ (bottom) and its ball-milled counterparts. The chemical shifts are referenced to the ^6^Li signal in CH_3_COOLi·2H_2_O. With both increasing milling time and rotational speed, a new NMR signal appears at *ca.* 0.6 ppm. It continuously gains in intensity and starts to dominate the spectrum of the sample that was milled for 30 min at a rotational speed of 400 rpm. A further shift of the whole signal toward 0.75 ppm is seen for even longer milling times (60 min, 120 min, and 400 rpm). We attribute this NMR line to the formation of amorphous LGPS. The line at 0.93 ppm refers to an impurity such as orthorhombic LGPS.

The corresponding ^6^Li MAS NMR spectra of crystalline LGPS and of the samples subjected to high-energy ball-milling are shown in Figure 4. A deconvolution of the ^6^Li spectra and the respective fit parameters can be found in the Supporting Information (Figure S5 and Table S5). The spectrum of crystalline LGPS is mainly composed of a single line, which represents an average signal due to fast Li^+^ hopping processes between the magnetically inequivalent sites in LGPS. Most importantly, upon milling, a new NMR line emerges, which we attribute to amorphous LGPS. It is worth noting that also this line is a motionally averaged one. We see that the change in average chemical shift values of the Li spins in the two distinct phases, 0.5 ppm (crystalline) *versus* 0.6 ppm (amorphous phase, short milling periods), differs only slightly. Despite these small changes, the ^6^Li MAS spectra confirm the results of our ^31^P NMR MAS experiments. In addition to ^31^P MAS NMR, the final shift of the ^6^Li MAS NMR lines toward 0.75 ppm for long milling times reveals further changes either in the local (distorted) structure or in ion dynamics for these samples.

It is worth noting that the shallow line at 0.93 ppm shows a small amount of Li spins in a different magnetic environment. This line cannot be attributed to β-L_i3_PS_4_ as the ^6^Li MAS NMR line of an in-house reference shows a signal at 0.76 ppm; see Figure S7. More likely, it reflects orthorhombic LGPS being detectable also for the sample milled for 15 min at 300 rpm (see Figure S5). The signal broadens upon milling and becomes almost no longer detectable for heavily milled samples, as also seen for the corresponding line in ^31^P NMR. The presence of a small amount of orthorhombic LGPS from the beginning would mean that all phases have the same chemical stoichiometry. Furthermore, it would support our findings for having no particular evidence, so far, that the amorphous phase strongly deviates in chemical composition from that of unmilled LGPS.

Finally, NMR, and to a certain degree XRD as well, helped us in visualizing the change in local disorder upon mechanical treatment. Defects and Li site disorder, polyhedra distortions, as well as the generation of strain lead to significant changes in ^31^P and ^6^Li MAS NMR spectra; see also Figure S6 and Table S6. Under the conditions of soft mechanical treatment, we suppose that a core–shell structure is generated with the amorphous phase covering the (nano-)crystalline LGPS regions. This picture resembles that of nanocrystalline alumosilicates and nanoglasses obtained after mechanical treatment.^54^

The amount of crystalline regions drastically reduces if we increase the milling time to 60 or 120 min at a rotational speed of 400 rpm. According to ^31^P MAS NMR, the spectra suggest that approximately >80% of the amorphous material is produced under these milling conditions; see the corresponding spectra in Figure 3b. Hence, these samples have to be described as being a mixture of two phases with a small amount of LGPS nanocrystallites being embedded in an amorphous matrix.^74^ This view is also supported by X-ray powder diffraction, as mentioned above.

### 3.2 Ion Dynamics as Seen by Conductivity Spectroscopy

**Figure 5:**
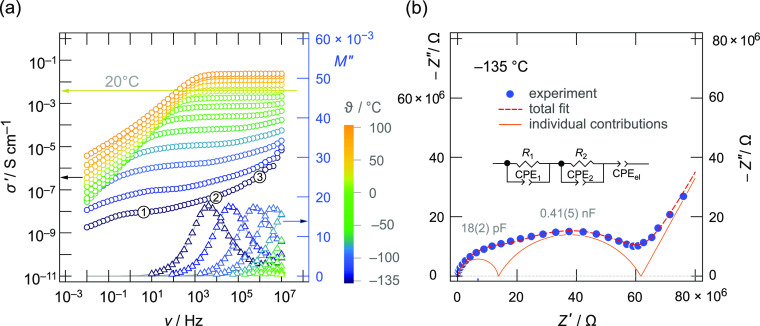
(a) Conductivity isotherms of microcrystalline Li_10_GeP_2_S_12_ recorded over broad ranges in temperature and frequency. At 20 °C, the prominent DC plateau indicates an (overall) electric conductivity of 3.9 mS cm^–1^. For comparison, the ionic conductivity at −60 °C reduces to 6 × 10^–5^ S cm^–1^. Additionally, *M*″(ν) spectra are shown whose apex frequencies roughly point to the centers of the high-frequency plateaus (bulk response) in σ′(ν) that is only recognizable at low temperatures. (b) Impedance plot −*Z*″(*Z*′) of the frequency-dependent (AC) impedance of microcrystalline Li_10_GeP_2_S_12_ measured at −135 °C. The two semicircles were parameterized with an appropriate equivalent circuit consisting of two *R*–CPE elements, each being composed of a resistor *R* connected in parallel with a constant phase element CPE, followed by a single CPE that represents electrode polarization at the lowest frequencies; see also Table S7. Capacitances indicate the bulk (18 pF) and the overall electric response (0.41 nF) that is influenced by the grain boundary regions of the thiophosphate. See text for further details.

Broadband impedance spectroscopy helped us to study the impact of structural disorder and downsizing the crystallite size on the overall Li^+^ ion dynamics. In Figure 5, the full electrical response of microcrystalline, that is, unmilled LGPS is shown by three presentations of the data collected. In Figure 5a, the so-called conductivity isotherms are displayed together with the modulus isotherms as spectroscopic plots.

Conductivity spectra are obtained by plotting the real part, σ′, of the complex ionic conductivity σ as a function of frequency ν. At high temperatures and low frequencies, the curves reveal a strong decay with decreasing frequency owing to polarization effects because of the ion-blocking electrode materials used to contact the sample. At sufficiently high temperatures, this polarization regime passes into a frequency independent plateau region, which we identify as the so-called DC (direct current) regime. Conductivity values of this regime directly mirror either bulk ion dynamics and/or ion transport that is affected by (ion-blocking) grain boundary regions. Indeed, at low temperatures, we observe a DC plateau in the regime of low frequencies (labeled 1 in Figure 5a) and another one being slightly inclined at higher frequencies (see label 2). At elevated *T* and using the σ′(ν) representation, the two processes cannot be separated any longer from each other. Plateau 2 finally passes into its dispersive (Jonscher-type) region (labeled 3) as it is best seen at low temperatures. At 20 °C, which is the temperature where only a single, but prominent DC plateau is seen, the specific (overall) conductivity of our microcrystalline sample turned out to be 3.9 mS cm^–1^. This value is only slightly lower compared to that presented in other studies.^24,35^ Most probably, small changes in sample preparation are the cause of this difference. Our value agrees, however, with those from other studies also investigating cold-pressed samples for their impedance measurements.^35^

Here, the two plateaus in σ′(ν) are assigned to the electrical responses of the bulk regions (plateau 2) and to the full electrical response (plateau 1) that slightly suffers from ion-blocking grain boundary regions. This assignment can be best understood when considering the corresponding Nyquist representation of the conductivity data. For this purpose, in Figure 5b, the complex plane plot is used to visualize the −*Z*″(*Z*′) location curve recorded at −135 °C. *Z*″ is the imaginary part, and *Z*′ is the real part of the complex impedance *Z*. The two (depressed) semicircles seen are to be characterized by capacitances *C* of 18 pF (bulk process) and 41 nF, respectively. Such values are clearly expected for a bulk response and a response that is influenced by grain boundary regions.^75^ To extract these capacitance values, we evaluated the complete location curve with an appropriate equivalent circuit composed of resistors and constant phase elements (CPEs) connected in parallel to represent each semicircle, details are also given in the Supporting Information in Table S7. Figure 5b shows the total fit and the individual components. Further Nyquist plots, which were recorded at higher temperatures, are shown in the Supporting Information. We observe that the grain boundary regions in a sulfide such as LGPS decrease the overall macroscopic conductivity of the samples but turned out to be much less blocking than that in oxide systems, for example.^56,76^

While at low temperatures the influence is measurable, at higher *T*, the resistive effect of the grain boundary regions on macroscopic transport is in many cases negligible. This observation is in agreement with the fact that in this high-*T* limit also, σ′(ν) does only reveal a single DC plateau. It is worth mentioning that the semicircle affected by Li^+^ transport across the grain boundary regions turned out to be clearly depressed. This feature is called non-Debye behavior and can be understood in terms of correlated motion or a distribution of electrical relaxation rates governing Li^+^ transport in conjunction with such regions. It goes along with the parameter *n*, characterizing the underlying CPE element which turned out to be significantly lower than 1; see Table S7.^77^ This observation is in contrast to the high-frequency semicircle, which shows that bulk ion dynamics is to be described by a narrower distribution function.

Alternatively, electric modulus spectra *M*″(ν) were evaluated to complement the electrical characterization of the microcrystalline sample; see Figure 5a. The electric modulus has the same physical interpretation as the imaginary part of the impedance *Z*″. *M*″ is proportional to the inverse complex permittivity ε. The amplitude of *M*″ is proportional to 1/*C*; thus, *M*″ is highly selective for processes that are to be characterized by low capacitances such as bulk processes.^75^ Hence, we expect that the *M*″(ν) curves are mainly governed by the electric bulk response. Indeed, the apex frequencies of the *M*″(ν) peaks often coincide with the beginning of the conductivity plateau that characterizes bulk properties. Therefore, this plateau was consequently assigned to the bulk response, that is, to the electrical response of the intragrain regions. The exact position of this plateau was determined from the maxima seen in tan(φ) with φ being the electric loss angle; see Figure S9.

**Figure 6:**
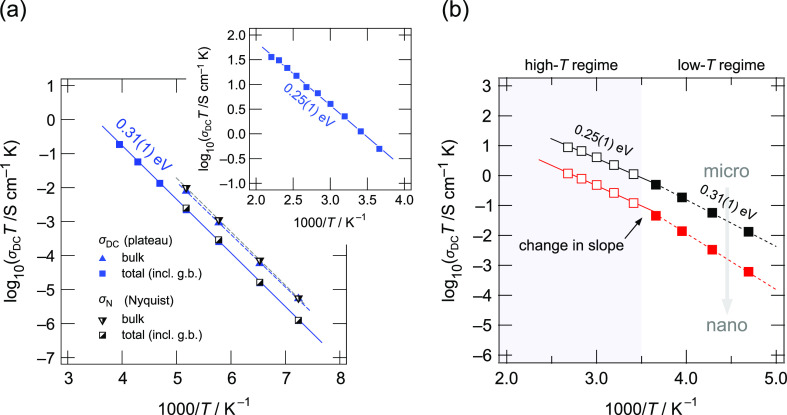
(a) Arrhenius diagram of microcrystalline LGPS showing the change of the bulk (intragrain) and the total (long-range) electrical conductivity either determined from the conductivity isotherms (σ_DC_) or extracted from the complex plane plots (σ_N_), that is, the Nyquist representation, in the temperature range from −135 to −80 °C. At temperatures higher than −80 °C, a separation of the two contributions is no longer possible. Lines represent fits with a linear function to determine the activation energies as indicated. At approximately −20 °C, the slope of the Arrhenius line changes. At elevated *T*, the activation energies decreased to 0.25 eV; see the inset. (b) Change of overall (total) conductivity of microcrystalline LGPS and ball-milled LGPS, which was treated for 120 min at 400 rpm. The dashed and solid lines are Arrhenius fits revealing the activation energies indicated. See text for further explanation.

To study the temperature dependence of the two relaxation processes, we evaluated (i) the values from the conductivity plateaus and (ii) analyzed the resistivity values extracted from parameterizing the curves of the Nyquist plots. The corresponding specific conductivities are shown in Figure 6. Values denoted with g.b. indicate those that take into account the resistive nature of the grain boundary regions. While the values characterizing overall electric properties in LGPS do coincide, only slight changes are seen for the values referring to bulk electrical relaxation. Within error limits, the resulting activation energies of the total and the bulk ion conductivities are the same (0.31 eV). Hence, the change, when going from bulk to overall properties, has to be looked for in a difference of the Arrhenius prefactor, which includes, for example, geometric effects, attempt frequencies, jump distances, and the migration entropy. In conductivity spectroscopy, it also contains the number fraction of charge carriers participating in the ionic conduction process. The value of 0.31 eV is in perfect agreement with that reported by Bron *et al.*(78)

In the inset of Figure 6a, the temperature dependence of the total conductivities is shown. We notice that the activation energy *E*_a_ decreases from 60 meV to 0.25 eV. This change, which occurs at −20 °C, is accompanied by a decrease of the corresponding Arrhenius prefactor. So far, the kink in Arrhenius behavior has also been discussed by others.^31,34,35^ Kuhn *et al.*(31) proposed that at lower *T*, the resistive nature of grain boundaries starts to influence σ, leading to a higher overall activation energy at temperatures well below ambient temperature. Here, we see that ionic transport involving grain boundaries does not increase *E*_a_ but rather affects the prefactor. As an alternative to earlier explanations, the change from 0.31 eV (at low *T*) toward 0.25 eV (at higher *T*) could also reflect a transition from correlated to less correlated motion. Such a transition has been used to explain similar kinks of Arrhenius lines in β-alumina,^79^*closo*-borates^80^ and metal organic frameworks^81^ with Na^+^ and Li^+^ ions as the main charge carriers.

As has been discussed in the literature additionally, Li^+^ ion migration in Li_10_GeP_2_S_12_ can be grouped into two general mechanisms, that is, cation transport along the *c*-direction and ion diffusion in the *ab*-plane of the crystal structure; see Figure S12.^29,32,33,82,83^ Based on computational calculations, Li^+^ transport along the *c*-direction was characterized by an activation energy of 0.17 eV. Ion dynamics in the *ab*-plane was, however, calculated to be governed by a hopping barrier of 0.28 eV.^82^ The latter value agrees very well with the activation energy probed in this study at lower *T* (0.31 eV). Hence, we conclude that the rate-limiting step for long-range ionic conduction presumably involves atomic jumps along this plane^32^ as these may circumvent blocking defects of the rapid 1D pathways along the *c*-direction. In this sense, the kink seen in the Arrhenius behavior might represent a change from quasi 1D transport toward 3D dynamics.

### 3.3 Ion Transport in Ball-Milled LGPS

**Figure 7:**
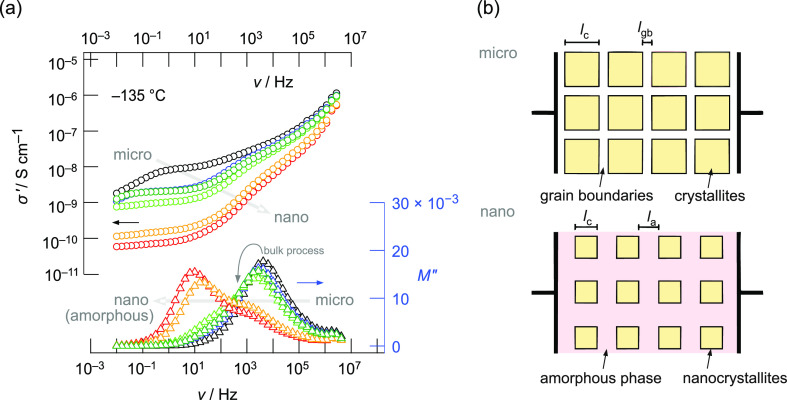
(a) Conductivity isotherms and electric modulus spectra of Li_10_GeP_2_S_12_ recorded at −135 °C. Data refer to micro- and nanocrystalline LGPS, that is, the ball-milled samples. Starting with microcrystalline LGPS (black), the curves refer to the following milling conditions: 15 min at 300 rpm (blue), 15 min at 400 rpm (dark green), 30 min at 400 rpm (bright green), 60 min at 400 rpm (yellow), and 120 min at 400 rpm (red). Ionic conductivity is clearly reduced upon milling. A second *M*″ peak at lower frequencies emerges, which we assign to the formation of an amorphous phase. (b) Brick-layer model used to illustrate the morphological changes taking place during milling. During mechanical milling, the crystallite size is reduced and the narrow grain boundaries are replaced by a thicker amorphous (inter)phase. *L*_c_, *l*_gb_, and *l*_a_ correspond to the thickness of the crystallites, grain boundaries, and amorphous phase, respectively.

In Figure 7a, the conductivity isotherms recorded at −135 °C of microcrystalline LGPS are compared with those from the milled samples. Isotherms measured at such a low temperature allow for the easiest discrimination of the bulk and grain boundary contributions, as the corresponding characteristic electrical relaxation frequencies are much lower than at room temperature. Surprisingly, we observe that upon mechanical treatment, the isotherms σ′(ν) shift toward lower conductivity values. Ball-milling does not lead to any further enhancement in ion dynamics in LGPS.

Simultaneously, we find two significant changes in electric modulus spectra, *M*″(ν), that perfectly mirror the overall changes in conductivity spectroscopy even better. First, after soft ball-milling the sulfide sample, the original modulus peak of crystalline LGPS shifts toward lower frequencies. As the apex frequency of the *M*″(ν) peak is proportional to the mean Li^+^ jump rate, also bulk ion dynamics decreases upon milling. Most likely, defects generated in the interior of the nanocrystallites hamper ionic transport. Second, a new peak appears upon milling that is located at lower frequencies pointing to a considerably slower electrical relaxation process in the ball-milled samples. At the beginning, that is, after short milling times, it manifests itself as a shoulder of the main peak. Mechanical treatment for 60 min at 400 rpm causes the new peak, however, to shift to even lower frequencies (10 Hz) and to visibly gain in intensity. This transformation of the macroscopic electrical response seen after 60 min of milling fully reflects the changes in local environments observed by the ^31^P and ^6^Li NMR nuclei on the angstrom length scale, *vide supra*.

Since the amplitudes of the two modulus peaks differ only by a factor of 2, the processes they reflect originate both from the bulk.^75^ While the original modulus peak characterizes intragrain ion dynamics (see above), we attribute the new one to the electrical relaxation to which the charge carriers are subjected to structurally disordered, amorphous LGPS. We notice that at −135 °C, the Li^+^ transport in the crystalline regions of LGPS, although being affected by ball-milling, is still by 2 orders of magnitude higher than that in the amorphous phase. Since this low conducting phase does also dominate the conductivity response σ′(ν) at low frequencies, see the plateaus at *ca.* 1 Hz, the former grain boundary response seen for microcrystalline LGPS (as discussed above) is almost masked.

The complete set of all conductivity spectra and Nyquist plots measured are provided in the Supporting Information (see Figures S8, S10, and S11). Table S8 lists the specific conductivities at 20 °C, the activation energies and capacitances that we obtained by analyzing the complex plane plots with appropriate electrical equivalent circuits. The possibility to separate individual components in the Nyquist representation depends on temperature. For the microcrystalline and for the 15 min milled samples, the crystalline response can still be separated from the total one. For the samples equipped with large amounts of the resistive, amorphous phase, such a separation was, however, fraught with difficulties as also the capacitances of the individual contributions to the full response were too similar to allow us to resolve the individual contributions.^75,84^

As is seen in the series of Nyquist plots shown in the Supporting Information (Figures S6), the amount of amorphous phase is mainly responsible for the decrease in overall ionic conductivity. Interestingly, the capacitances *C* describing its electrical response steadily decreases with milling time. Larger capacitances observed for the samples subjected to very soft milling, that is, for only 15 min at 300 rpm (0.14 nF) or 400 rpm (98 pF), correspond to *M*″ peaks with low intensity; see Figure 7a. Most likely, the electrical response of these softly treated LGPS samples consists of (i) overlapping contributions from grain boundary and amorphous regions or (ii) originates from a small fraction of amorphous phase in between or covering the (nano-)crystallite domains still having considerable grain boundary character.

Here, we tried to understand the evolution of the ion dynamics as probed by broadband conductivity with the brick-layer model,^75^ where the grain boundaries are replaced by a growing amorphous phase with prolonged milling; see Figure 7b. Assuming such a brick-layer model, the relative capacitances of bulk and grain boundaries can be calculated according to *C*_c_/*C*_gb_ = *l*_gb_/*l*_c_ describing the ratio of capacitances and geometric properties as indicated in Figure 7b.^75^ As milling proceeds, amorphous materials and nanocrystallites are formed. The amorphous phase will be generated mainly at the outer layers of the grains, that is, at the grain boundary regions.^85^ Consequently, due to this core–shell structure, the grain boundaries become spatially less well defined and are replaced by an amorphous region with an increased spacing *l*_a_ instead of *l*_gb_. Simultaneously, *l*_c_ reduces upon milling. Thus, this trend would explain the evolution of *M*″ with both increasing milling time and rotational speed: we observed higher capacitances of the (nano)crystalline bulk contributions (lower amplitudes of *M*″) and lower capacitances of the grain boundary regions and/or amorphous fractions (higher amplitudes of *M*″); see Figure 7a.

**Figure 8:**
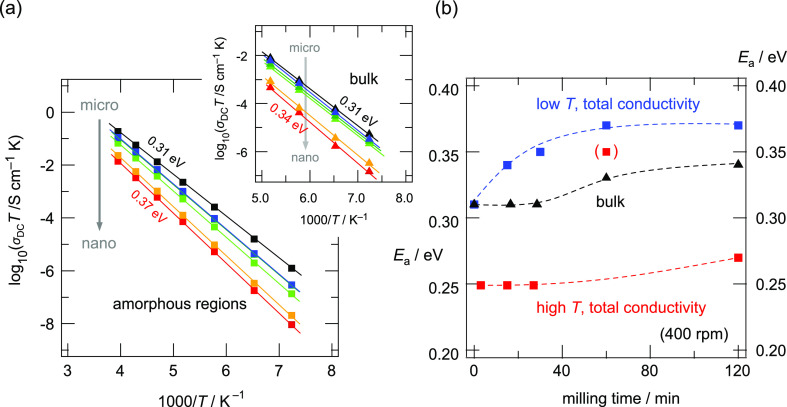
(a) Arrhenius representation of the temperature dependence of the ionic conductivity plotted as log_10_(σ_DC_*T*) against the inverse temperature expressed as 1000/*T*. Here, only the low temperature regime below −20 °C is shown. σ_DC_ values were directly read off from the plateaus of the corresponding conductivity isotherms. The data correspond to microcrystalline LGPS (black) and samples milled for 15 min at 300 rpm (blue), 15 min at 400 rpm (dark green), 30 min at 400 rpm (bright green), 60 min at 400 rpm (yellow), and 120 min at 400 rpm (red). At temperatures lower than or equal to −80 °C, the bulk and grain boundary contributions could be separated. The bulk response is shown in the inset. A decrease in activation energy is observed also for this process. (b) Activation energies derived from the linear fits for the bulk and grain boundary/amorphous responses. The latter was obtained for both the low (≤−20 °C) and high temperature regimes.

Until now, we proposed that the newly formed amorphous interphase in between the remaining nanocrystallites is responsible for the reduced overall ionic conductivity of the material. As mentioned above, a separation of the two contributions, that is, the intragrain response and the response from the amorphous phase, turned out to be difficult through the analysis of the Nyquist curves *via* equivalent circuits and for temperatures higher than room temperature. However, at temperatures below −80 °C, we were able to separate the responses by analyzing the different plateaus in the corresponding conductivity spectra with the help of the information from modulus spectroscopy. The resulting conductivities referring to the g.b./amorphous contribution are shown in Figure 8a, and the inset in Figure 8a shows the conductivities of the intragrain (bulk) regions. We recognize that with increasing ball-milling duration, the ionic conductivity that corresponds to the low-frequency plateau in Figure 7a clearly reduces. The corresponding activation energies increase from 0.31 eV to *ca.* 0.37 eV; see Figure 8b.

Importantly, as discussed above for data recorded at −135 °C, *vide supra*, ball-milling does also affect ion dynamics in the nanocrystalline regions. The inset of Figure 8a shows that intragrain Li^+^ hopping also reduces with increasing milling time. The activation energy follows this trend and slightly increases from 0.31 to 0.34 eV. This behavior does not support the proposed increase in ionic conductivity, as suggested by Dawson and Islam for nanocrystalline LGPS benefiting from local disorder, changes in local ion coordination or even a change in the dimensionality of the dynamic process.^42^ The simulations suggest that diffusion along the *ab*-plane, that is, in-plane diffusion, is facilitated when going from the bulk to nanocrystals with a grain volume of 10 nm^3^; hence, they postulate a change from 1D to 3D diffusion. Changes of the local Li^+^ environments are made responsible for this increase observed; in particular, they observed a decrease of the Li–Li and Li–S coordination numbers by one at a distance of >5 Å; below 4.5 Å, any such change turned out to be rather small.^42^ Here, the defects introduced during high-energy ball-milling clearly hamper long-range ion transport. We conclude that the crystallographically well-defined diffusion pathways in LGPS become distorted or even blocked by the defects introduced into the crystalline regions.

In summary, the effect of ball-milling on overall ion dynamics in LGPS is twofold: (i) amorphous LGPS is detrimental for facile long-range ion transport and (ii) disorder and distortions do not promote intragrain ion dynamics in the nanocrystalline regions. Altogether, as compared to unmilled LGPS, the conductivity of a sample that has been milled for 120 min turned out to be lower by roughly 1 order of magnitude at 20 °C (0.41 mS cm^–1^).

Finally, we will look at the change of total ion conductivity measured over the whole temperature range accessible with our experimental setup. Coming back to Figure 6b, the specific conductivities of the ball-milled sample refer to the low-frequency region of the corresponding conductivity isotherms. As for the microcrystalline sample, a kink in the Arrhenius line is seen for the LGPS sample milled for 120 min (400 rpm); see also Figure S13 for the data of all samples. This kink supports our assumption that it is not simply related to a grain boundary effect, as proposed earlier,^31^ as the response of the ball-milled sample is largely governed by the amorphous regions. Likewise, a change in dimensionality of the transport process could hardly serve as an argument to understand this kink as it is also seen for a mostly disordered sample. Instead, it could indeed reflect a change from correlated to uncorrelated motion that is triggered by temperature, as suggested above.

## 4 Conclusions

Li_10_GeP_2_S_12_ (LGPS) is known as a highly conducting solid electrolyte that pushed open a door to explore similar structures and other classes of materials to study their ion transport properties. There has been an ongoing debate in the literature as to what factors drive the exceptionally high ionic conductivity in LGPS. The introduction of structural disorder and nanosize effects being beneficial for many poor ionic conductors has, so far, not been studied experimentally for LGPS. To contribute in an experimental approach, we employed high-energy ball-milling to reduce the crystallite size of solid-state reaction-synthesized LGPS and investigated Li^+^ dynamics. Here, we showed that nanosizing and disorder, if realized through high-energy ball-milling, do decrease Li^+^ ion dynamics in LGPS. Ball-milling leads to the formation of nanocrystallites next to structurally amorphous regions. Local distortions, as sensed by ^31^P and ^6^Li high-resolution NMR, seem to block ion transport not only in the amorphous phase but also in the defect-rich nanocrystalline regions generated. The latter finding was revealed by applying low-temperature broadband conductivity spectroscopy, which enabled us to investigate the bulk response independently from that characterizing the dynamic properties of the amorphous regions.

Obviously, in materials with crystallograpically well-defined diffusion or transport pathways, the introduction of higher dimensional defects is detrimental for fast ion dynamics. Such defect structures hinder the ions to be guided through the crystal structure on a long-range length scale. Our results emphasize the importance of synthesizing pure and crystalline phases for materials like LGPS that provide (low-dimensional) rapid migration pathways formed by their partially filled Li-sublattices. In LGPS, overall ion dynamics turned out to be sensitively dependent on structural disorder. Hence, a proper control of the defect chemistry and the defect concentration represents key factors to understand and successfully manipulate ion dynamics in materials with high ionic conductivities.

## Supporting Information Available

Details on the MAS NMR acquisition parameters; deconvolution and fitting parameters; PXRD pattern of microcrystalline LGPS and details on Rietveld refinement; Raman spectra the LGPS samples; further Nyquist plots and conductivity spectra; results from evaluating the impedance data; and further structural illustrations highlighting the conduction pathways in LGPS (PDF)

## Abbreviations

LGPS: Li_10_GeP_2_S_12_
MAS NMR: magic angle spinning nuclear magnetic resonance
PXRD: powder X-ray diffraction
