# Supplementary material for: Ionic Conductivity of Nanocrystalline and Amorphous Li10GeP2S12: The Detrimental Impact of Local Disorder on Ion Transport
Source: J Am Chem Soc. 2022 May 24;144(22):9597–609. doi: 10.1021/jacs.1c13477 (PMC9185751; doi:10.1021/jacs.1c13477)
Supplement: Supplementary file 1 — ja1c13477_si_001.pdf [file ja1c13477_si_001.pdf]

Supporting Information

Ionic Conductivity of Nanocrystalline and Amorphous $Li_{10}GeP_2S_{12}$: The Detrimental Impact of Local Disorder on Ion Transport

Lukas Schweiger,[1] Katharina Hogrefe[1]*, Bernhard Gadermaier[1], Jennifer L. M. Rupp[2,3], and H. Martin R. Wilkening[1]

[1] Institute for Chemistry and Technology of Materials, Christian Doppler Laboratory for Lithium Batteries, Graz University of Technology (NAWI Graz), 8010 Graz, Austria

[2] Electrochemical Materials, Department of Materials Science and Engineering, Massachusetts Institute of Technology, Cambridge, MA 02139, USA.

[3] Electrochemical Materials, Department of Electrical Engineering & Computer Science, Massachusetts Institute of Technology, Cambridge, MA 02139, USA

Table S1. Overview of LGPS and its variants presented in literature including the starting materials, details on the synthesis procedure and ionic conductivities measured by impedance spectroscopy (measured at temperature *T*).

composition	starting materials	Preparation step, temperature, time	σ / mS cm^{-1} (T / K)
$Li_{10}GeP_2S_{12}$[1]	Li_2S, P_2S_5, GeS_2	vibration milling, sealed in quartz tube, 550 °C for 8 h	12 (300)
$Li_{10}GeP_2S_{12}$[2]	Li_2S, P, Ge, S	ball milling, sealed in quartz tube, 420 °C for 24 h	9 (298)
$Li_{10.05}Ge_{1.05}P_{1.95}S_{12}$[3]	Li_2S, P_2S_5, GeS_2	vibrating mill, tube furnace under argon flow, 550 °C for 8 h	6.85 (298)
$Li_{10}GeP_2S_{12}$[4]	Li_2S, P_2S_5, GeS_2	$Li_{10}GeP_2S_{12}$ synthesized by solid-state reaction, dissolved/dispersed in methanol, drying and subsequent annealing (550 °C for 8 h)	1.7 (298)
$Li_{9.6}P_3S_{12}$[5]	Li_2, P_2S_5, P	ball milled, sealed in quartz tube, heated between 230 °C and 260 °C for 4 h	1.20 (298)
$Li_{11}Si_2PS_{12}$[6]	Li_2S, P, Si, S	ball milling, sealed in quartz tube, 550 °C for 96 h, high-pressure treatment at 450 °C, 3 GPa < p < 5 GPa	4 (297)
$Li_{10}SnP_2S_{12}$[7]	Li_2S, P_2S_5, Li_4SnS_4	mixing and grounding with agate mortar, sealed in quartz tube, 600 °C for 48 h	4 (300)
$Li_{9.54}Si_{1.74}P_{1.44}S_{11.7}Cl_{0.3}$[5]	Li_2S, P_2S_5, GeS_2, SiS_2, LiCl	ball milled, sealed in quartz tube, 475 °C for 8 h	25 (298)
$Li_{10}GeP_2S_{12-x}O_x$[8]	Li_2S, Li_2O, P_2S_5, GeS_2	ball milled, sealed in quartz tube, 550 °C for 24 h	10.3 for x = 0.3 (298) 8.43 for x = 0.6 (298)
$Li_{10}SiP_2S_{12-x}O_x$[9]	Li_2S, P_2O_5, P_2S_5, SiO_2, SiS_2	ball milled, sealed in quartz tube, 550 °C for 48 h	3.1 for x = 0.7 (298)

Rietveld refinement of the X-ray diffraction data

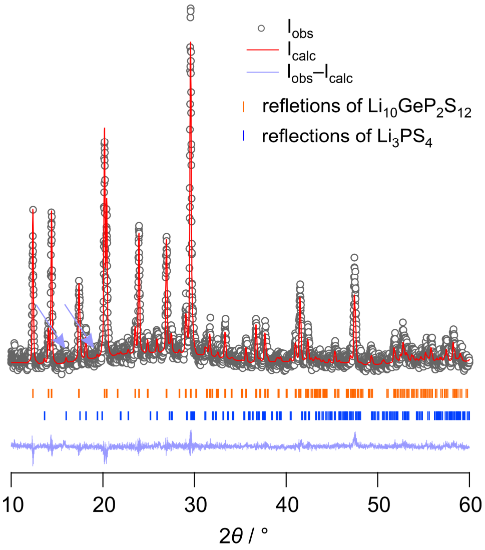

Figure S1. Rietveld refinement of the PXRD pattern of $Li_{10}GeP_2S_{12}$ recorded at 293 K. The pattern was recorded with a Rigaku SmartLab using a step size of 0.01° and an acquisition speed of 2 °/min. The observed and calculated profiles are shown together with the Bragg positions of the identified phases. The pattern allowed for a qualitative phase identification and reveals that the synthesis of $Li_{10}GeP_2S_{12}$ was successful but with the minor phase β-Li_3PS_4 or orthorhombic LGPS being present in the sample; refinement shown for assuming Li_3PS_4. No quantitative interpretation and calculation of phase fractions was attempted. The refinement was based on the crystal structure reported by Kuhn *et al.*[10] The R_{wp} (weighted profile factor) profile is 19.74 % and the GoF (goodness of fit) amounts to 4.52.

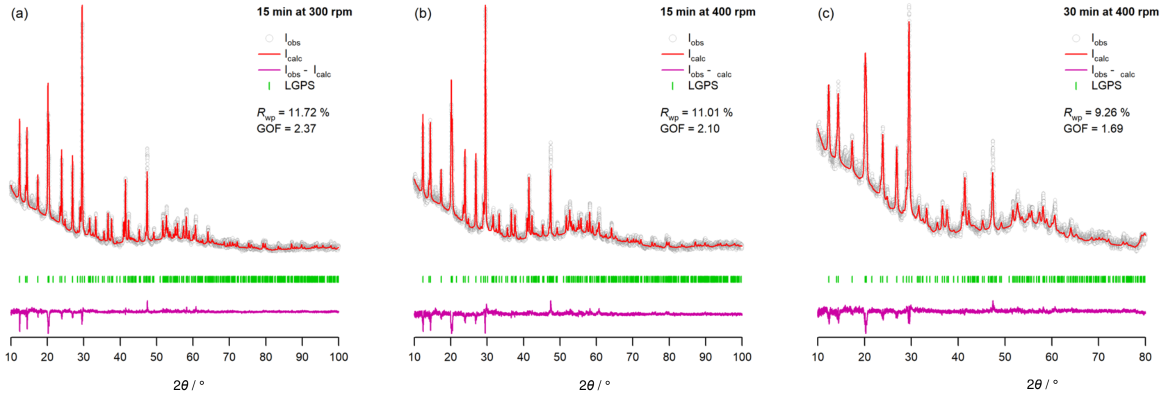

Figure S2. Rietveld refinements of the PXRD patterns of $Li_{10}GeP_2S_{12}$ recorded at 293 K; (a) 15 min at 300 rpm, (b) 15 min at 400 rpm and (c) 30 min at 400 rpm. The patterns were recorded with a Rigaku MiniFlex using a step size of 0.005° and an acquisition speed of 1 ° min^{-1}. The observed and calculated profiles are shown together with the Bragg positions of $Li_{10}GeP_2S_{12}$. The R_{wp} (weighted profile factor) profiles and the GoFs (goodness of fit) are presented in the respective figures.

Rietveld refinement. Rietveld refinements were performed for microcrystalline as well as for all nanocrystalline samples. All refinements yielded adequate GoF values, showing that in all cases $Li_{10}GeP_2S_{12}$ could be identified unambiguously. However, Rietveld refinement was not regarded reasonable for the (mainly amorphous) samples milled for 60 rpm or 120 min. Since these spectra contained broad and few well resolved reflections, refinements suffer from background noise.

Performing an estimation of the crystallite size by Rietveld refinement confirmed the trend already observed, with the calculated crystallite sizes being in the nm regime for the milled samples and decreasing with prolonged milling.

Estimation of the mean crystallite size

The fwhm of the peak at 29.5 ° was used to estimate the crystallite size (coherent scattering domain) with the Scherrer equation.[11]

$$d = \frac{K\lambda}{\Delta \cos\theta}$$

d is the crystallize size, *K* a constant set to 0.9, λ the wavelength, Δ the fwhm and θ the position of the peak analyzed. Here, no standard was used to quantify the instrumental broadening. Micro strain leads to a Gaussian peak shape, a reduced crystallite size will, however, mainly result in a Lorentzian one.[12] A Voigt function, *i.e.*, a convolution of Gaussian and Lorentzian functions, was used to analyze the reflection at 29.5°. After a milling time of 120 min (400 rpm) this reflection became very broad and was mainly of Lorentzian shape. Therefore, we assume that the width of the reflection is dominated by the reduced crystallite size. Hence, to approximate the crystallite size, instrumental broadening and broadening effects from micro strain were neglected. (It should be noted that both methods for determining the crystallite sizes (output from the Rietveld refinement and direct calculation using the Scherrer equation) should only be considered as approximations since no calibrated instrument or standards were using in this investigation.)

Raman spectroscopy

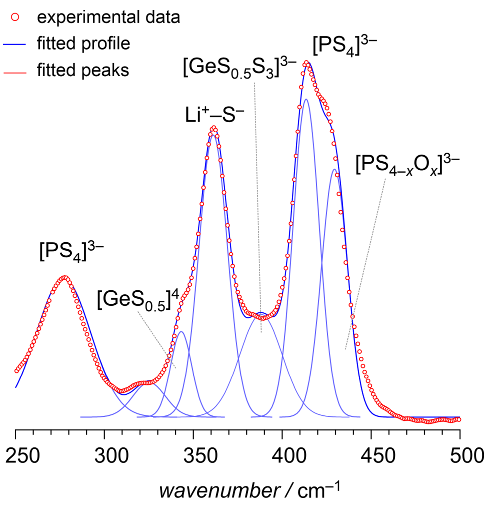

Figure S3. Raman spectrum of $Li_{10}GeP_2S_{12}$ obtained with a 532 nm Laser source (5 mW, 2.1 µm spot size, 2 s exposure time) and an Olympus objective (10-fold magnification); 60 spectra were recorded for each spot and accumulated. The estimated spectral resolution was between 5.5 cm^{-1} and 8.3 cm^{-1}. Measurements at (at least) two spots on the sample were acquired to be representative. The spectrum was analyzed by fitting Gaussian functions to the profile. The individual Gaussians are plotted in red, the resulting fit profile in blue and the residuals in magenta. The spectrum allowed for a complementary chemical characterization and confirmed the successful synthesis of $Li_{10}GeP_2S_{12}$. The assigned vibrational modes according to Hassoun *et al.*[13] are also indicated for each peak. A band that might be related to oxygen dissolved in $Li_{10}GeP_2S_{12}$ can be seen in the spectrum at around 435 cm^{-1}.[8]

^{6}Li and ^{31}P MAS NMR, acquisition parameters

Table S2. Acquisition parameters used to record the ^{6}Li and ^{31}P MAS NMR spectra

nucleus	pulse	recycle delay	scans
^{6}Li	3–4.5 μs (100 W)	10 s	64–128
^{31}P	1.35–1.45 μs (180 W)	10 s	64–128

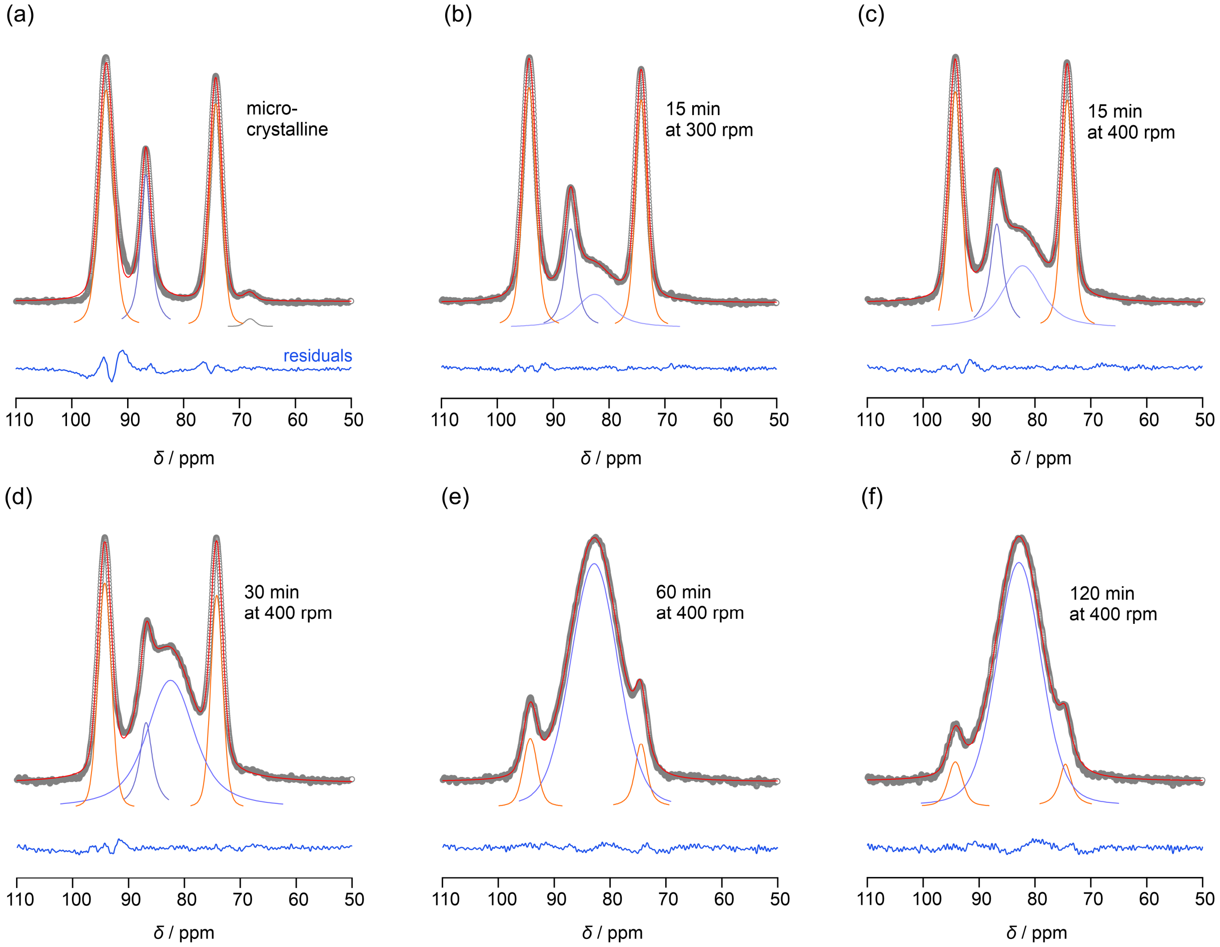

Figure S4. Deconvolution of the ^{31}P MAS NMR spectra of $Li_{10}GeP_2S_{12}$; (a) microcrystalline (unmilled) LGPS, (b) 15 min at 300 rpm, (c) 15 min at 400 rpm, (d) 30 min at 400 rpm, (e) 60 min at 400 rpm and (f) 120 min at 400 rpm. The spectra were recorded at 202.5 MHz and at a spinning speed of 25 kHz (2.5 mm rotors). All spectra were referenced to the ^{31}P MAS NMR signal of $CaHPO_4$. We used Voigt functions to parameterize the overall spectra, see solid lines.

Table S3. Parameters used to approximate the ^{31}P MAS NMR spectra and used for the calculation of the respective phase fractions. Note that the width does not refer to the full width at half height, it denotes a relative value

(a) microcrystalline	phase	width (relative values)	position / ppm	area (relative values)
	LGPS	0.74(1)	74.26(1)	0.328(2)
	side phase	2.0(2)	86.75(1)	0.244(2)
	LGPS	0.70(1)	93.86(1)	0.428(3)
(b) 15 min at 300 rpm	phase	width (relative values)	position / ppm	area (relative values)
	LGPS	0.85(4)	74.25(1)	0.314(1)
	amorphous	0.40(2)	82.37(3)	0.143(4)
	side phase	1.76(8)	86.86(1)	0.176(2)
	LGPS	0.77(1)	94.31(1)	0.367(1)
(c) 15 min at 400 rpm	phase	width (relative values)	position / ppm	area (relative values)
	LGPS	0.79(1)	74.22(1)	0.269(2)
	amorphous	0.33(1)	82.24(3)	0.263(5)
	aide phase	5(3)	86.83(1)	0.173(3)
	LGPS	0.72(1)	94.26(1)	0.295(1)
(d) 30 min at 400 rpm	phase	width (relative values)	position / ppm	area (relative values)
	LGPS	0.79(1)	74.22(1)	0.191(2)
	amorphous	0.20(1)	83.23(3)	0.560(7)
	side phase	0.84(4)	86.85(1)	0.039(3)
	LGPS	0.64(1)	94.28(1)	0.210(2)
(e) 60 min at 400 rpm	phase	width (relative values)	position / ppm	area (relative values)
	LGPS	0.68(2)	74.32(1)	0.047(2)
	amorphous	0.18(1)	82.75(1)	0.871(3)
	LGPS	0.70(2)	94.30(1)	0.082(2)
(f) 120 min at 400 rpm	phase	width (relative values)	position / ppm	area (relative values)
	LGPS	0.68(3)	74.34(1)	0.032(2)
	amorphous	0.19(1)	82.72(1)	0.909(4)
	LGPS	0.69(3)	94.28(1)	0.060(2)

Table S4. Phase fractions of $Li_{10}GeP_2S_{12}$. The side phase (87 ppm) and the amount of the amorphous phase as calculated from the ^{31}P MAS NMR peak areas. The phase fractions of the side phase were calculated assuming the stoichiometry of both Li_3PS_4 (white column) and a side phase with the same P-content as $Li_{10}GeP_2S_{12}$ (grey column), which would also correspond to an orthorhombic modification of LGPS

sample	$Li_{10}GeP_2S_{12}$ / wt%		side phase / wt%		amorphous phase / wt%	
microcrystalline (annealed)	84(1)	75.6(3)	16.4(3)	20.4(2)	–	–
15 min at 300 rpm	73.0(7)	67.1(1)	11.6(2)	17.6(2)	15.4(6)	14.3(4)
15 min at 400 rpm	60.5(8)	56.5(2)	11.3(3)	17.3(3)	28.2(8)	26.2(5)
30 min at 400 rpm	40.7(7)	40.0(2)	2.4(5)	3.8(3)	57(1)	55.9(7)
60 min at 400 rpm	12.9(3)	12.9(3)	–	–	87.1(6)	87.1(6)
120 min at 400 rpm	9.1(4)	9.1(4)	–	–	90.9(7)	90.9(7)

^{31}P MAS NMR. The peaks in the ^{31}P MAS NMR spectrum were fitted with Voigt functions and the peak area was determined. Comparing the peak areas, considering the different relative P contents of $Li_{10}GeP_2S_{12}$ and Li_3PS_4, allowed the determination of the phase fractions in weight percent, wt%. using the following formula:

$$\mathrm{wt\%}_a = \left(\frac{A_\mathrm{a}}{A_\mathrm{tot}}\frac{1}{x}\mathrm{MW_a}\right)\left(\sum_{i=1}^{j}\frac{A_i}{A_\mathrm{tot}}\frac{1}{x}\mathrm{MW}_i\right)^{-1}$$

A_a denotes the summed-up area of all peaks associated with a certain phase a, A_tot is the total area of all peaks (neglecting the minor peaks at 67 ppm and 36 ppm) and MW_a denotes the molecular weight of the respective phase a. x is a correction factor taking into account the different P contents of the phases (equals P in the chemical formula of that phase, *i.e.*, 1 for β-Li_3PS_4 and 2 two for LGPS). The right term is the same as the left term, but summing up over all phases j (being two in this case).

^{6}Li MAS NMR. Considering the ^6Li MAS NMR spectra shown in Figure 4. we see that the overall signal is composed of two lines appearing at chemical shifts of 0.93 ppm and 0.47 ppm. Analyzing the lines with Voigt functions yielded areas of 88 % and 12 % with respect to the total area under the signal. The main line at 0.47 ppm agrees very well with literature data.[14] The NMR line at 0.93 ppm cannot be attributed to β-Li_3PS_4 as the ^6Li MAS NMR line of an in-house β-Li_3PS_4 shows a signal at 0.76 ppm, see Figure S7. The small Li signal at 0.93 ppm might also stem from orthorhombic LGPS. The side phase is also detectable for the sample milled for 15 min at 300 rpm but is prone to amorphization upon more intensive milling.

Striking is the extremely low width (fwhm) of the line at 0.47 ppm of only 7.15 Hz. This value already indicates very fast Li-ion motional processes taking place in the sample at room temperature as expected for LGPS.[19]

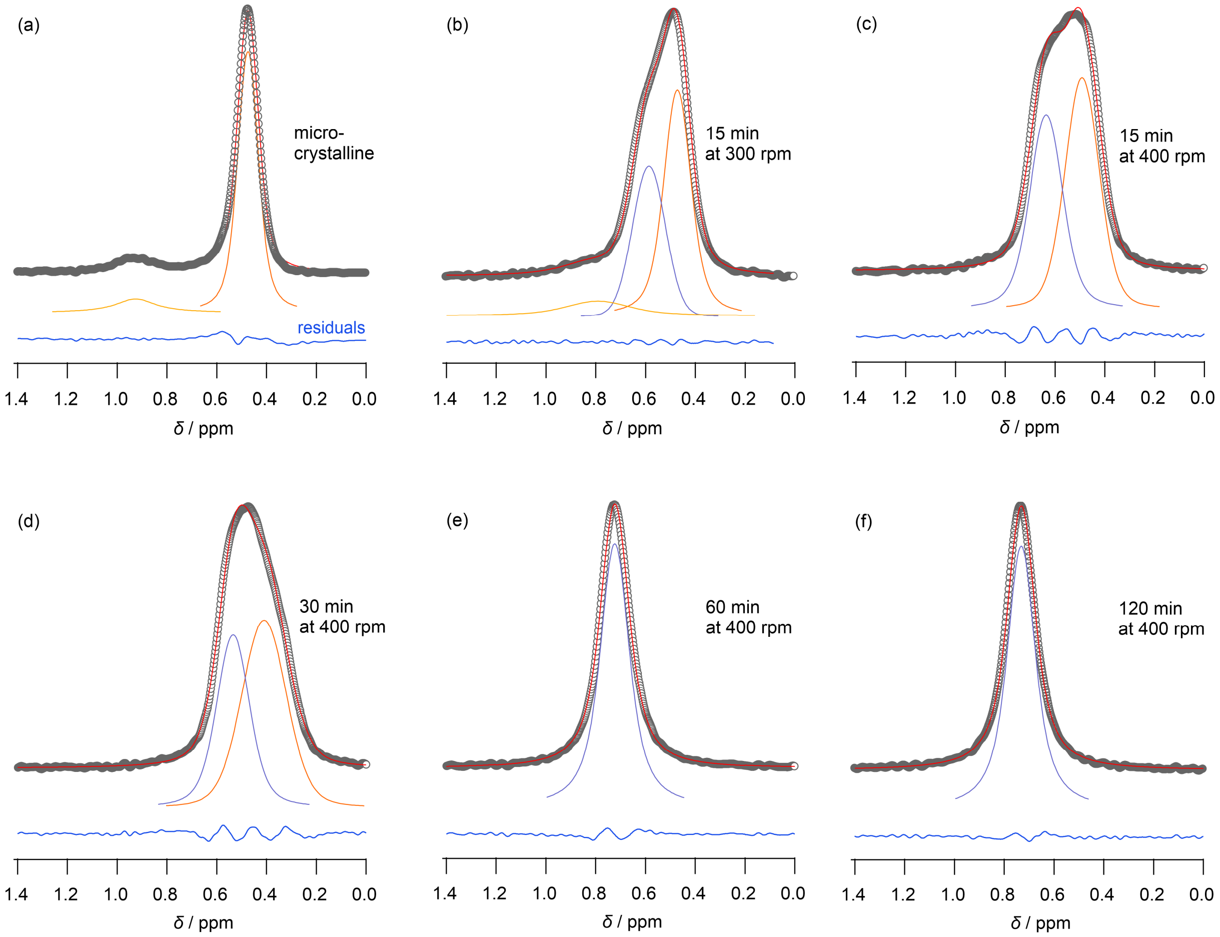

Figure S5. Deconvolution of the ^{6}Li MAS NMR spectra of $Li_{10}GeP_2S_{12}$; (a) microcrystalline (unmilled) LGPS, (b) 15 min at 300 rpm, (c) 15 min at 400 rpm, (d) 30 min at 400 rpm, (e) 60 min at 400 rpm and (f) 120 min at 400 rpm. The spectra were recorded at 73.6 MHz MHz and at a spinning speed of 25 kHz (2.5 mm rotors). All spectra were referenced to the ^{6}Li NMR signal of $H_3CCOOLi \cdot 2H_2O$. We used Voigt functions to parameterize the overall spectra, see solid orange lines. The total fit is indicated in blue, the residuals in magenta.

Table S5. Fit parameters obtained by fitting the ^{6}Li MAS NMR spectra. The peak enumeration is from right to left, that is, from low to high chemical shifts; note that the widths do not refer to the full width at half height; they denote relative values to compare the lines of each individual sample.

(a) microcrystalline		width (relative values)	position / ppm	area (relative values)
	peak 1	22.97(7)	0.47(1)	0.886(1)
	peak 2	52(33)	0.93(1)	0.114(1)
(b) 15 min at 300 rpm		width (relative values)	position / ppm	area (relative values)
	peak 1	17.5(5)	0.47(1)	0.553(7)
	peak 2	11(1)	0.59(1)	0.353(2)
	peak 3	9(5)	0.79(5)	0.094(9)
(c) 15 min at 400 rpm		width (relative values)	position / ppm	area (relative values)
	peak 1	11.7(2)	0.49(1)	0.539(2)
	peak 2	13.3(3)	0.64(1)	0.461(2)
(d) 30 min at 400 rpm		width (relative values)	position / ppm	area (relative values)
	peak 1	8.84(9)	0.41(1)	0.578(2)
	peak 2	13.5(2)	0.53(1)	0.422(2)
(e) 60 min at 400 rpm		width (relative values)	position / ppm	area (relative values)
	peak 1	22.0(3)	0.72(1)	1
(f) 120 min at 400 rpm		width (relative values)	position / ppm	area (relative values)
	peak 1	24.5(3)	0.73(1)	1

Table S6 and Figure S6 show the evolution of the MAS NMR line width with milling time. For the ^{31}P line widths, a slight increase in the line width for the two lines corresponding to the P sites in crystalline LGPS is observed. This slight broadening is a result of the introduction of defects and strain into the LGPS crystallites via the milling step. For the side phase characterized by the signal at 87 ppm, the line width shows no clear trend. Upon the formation of the amorphous phase the two ^{31}P NMR lines do overlap hindering a clear evaluation of the line width. Possibly, this line merges with the other lines or disappears. For the narrower ^{6}Li NMR line, we also see a small increase for the line width of the crystalline contribution resulting from the structural disorder introduced by the mechanical treatment. Interestingly, also for the ^{6}Li spectra an amorphous phase is observed, which first appears as a broad shoulder with downfield shift, see Figure 4.

According to the MultiPeak Fit analysis (IGORPro wavemetrics software) this signal is broader also pointing to a structurally less ordered structure. As two contribution to the ^{6}Li signal can clearly be observed for LGPS samples milled at 15 to 30 min. NMR clearly senses here two spin reservoirs with access to differently fast exchange processes. For the samples milled at longer times and showing an amorphous phase fraction > 80 wt% the ^{6}Li lines averages to only one signal, meaning that the Li-Li exchange in the sample is similarly fast for all spins.

Table S6. Line widths of individual NMR resonances derived from a MultiPeak Fit analysis (IGORPro wavemetrics software). For the ^{31}P NMR spectra we observed two signals from crystalline $Li_{10}GeP_2S_{12}$ next so a side phase and an amorphous phase, which forms upon milling. In the case of ^{6}Li MAS NMR, we followed the evolution of an amorphous phase upon milling, seen as a shoulder of the signal belonging to crystalline $Li_{10}GeP_2S_{12}$. Note that for long milling times only a single ^{6}Li signal is seen, which we attribute to a (mean) local chemical environment with amorphous character.

milling time / min	LGPS1 ^{31}P / ppm	LGPS2 ^{31}P / ppm	side phase ^{31}P / ppm	amorph. phase ^{31}P / ppm	LGPS ^{6}Li / ppm	amorph. phase ^{6}Li / ppm
0	2.62	3.06	2.20	-	0.10	-
15 at 300 rpm	2.44	2.70	2.55	6.97	0.13	0.15
15 at 400 rpm	2.46	2.72	2.78	8.09	0.16	0.16
30 at 400 rpm	2.49	2.79	2.04	10.89	0.21	0.16
60 at 400 rpm	2.44	2.98	-	10.00	-	0.14
120 at 400 rpm	2.46	3.09	-	9.68	-	0.13

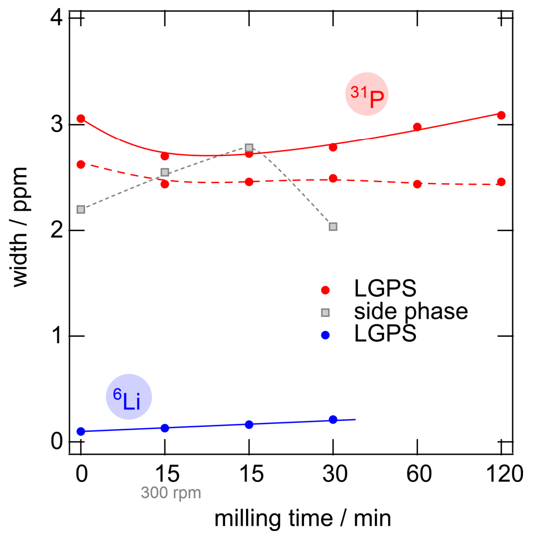

Figure S6. Evolution of the MAS NMR line width for the ^{31}P and ^{6}Li NMR spectra with milling time. Note that crystalline $Li_{10}GeP_2S_{12}$ has two magnetically inequivalent P sites. The crystalline part in the ^{6}Li NMR can only be distinguished up to a milling time of 30 minutes. The evolution of the line width of the amorphous phase fractions is shown in Table S6.

To ensure that all spins were detected upon acquisition, the MAS NMR experiments were carried out using different delay times between the scans. The corresponding spectra are shown in Figure S7. No differences in the spectra were observed neither for ^{6}Li nor ^{31}P also at very long delays of 1285 s and 450 s, respectively. Accordingly, quantification of the different phases was carried out using the spectra recorded with a recycle delay of 10 s.

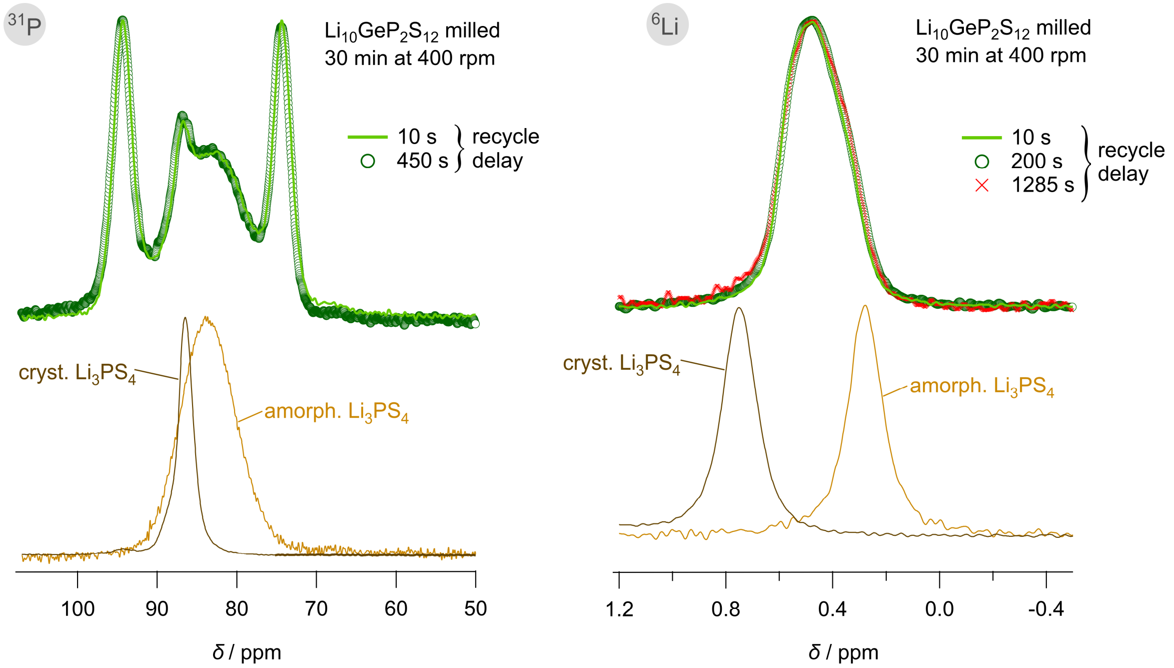

Figure S7. ^{31}P (left) and ^{6}Li (right) MAS NMR spectra of the LGPS sample milled for 30 minutes at 400 rpm (top) compared with the spectra of our in-house prepared references of crystalline and amorphous Li_3PS_4 (bottom). The NMR spectra were acquired using variable waiting times ranging from 10 s to values as high as 1285 s in order to detected also NMR components relaxing very slowly. Here, no differences show up suggesting that a recycle delay of 10 s is enough to detect all ^{6}Li and ^{31}P spins.

Impedance analysis

Table S7. Parameters obtained by fitting the equivalent circuit seen in figure 5, *i.e.*, the Nyquist representation of the impedance response of $Li_{10}GeP_2S_{12}$ at a temperature of – 135 °C.

contribution	R / Ω [a]	C of the CPE / F [b]	n of the CPE [c]	τ / s [d]
bulk	1.4×10^{7}	1.8×10^{-11}	0.89	2.5×10^{-4}
grain boundary	4.8×10^{7}	4.1×10^{-10}	0.67	2.0×10^{-2}
electrode	–	2.2×10^{-8}	0.68	–

[a] each resistor is associated with a resistance R.
[b] each CPE is associated with the capacitance C and the dimensionless parameter n.
[c] n indicates the deviation from perfect behavior of a capacitor ($n = 1$).
[d] for each semicircle, the associated time constant can be calculated by the relation $RC = \tau$.

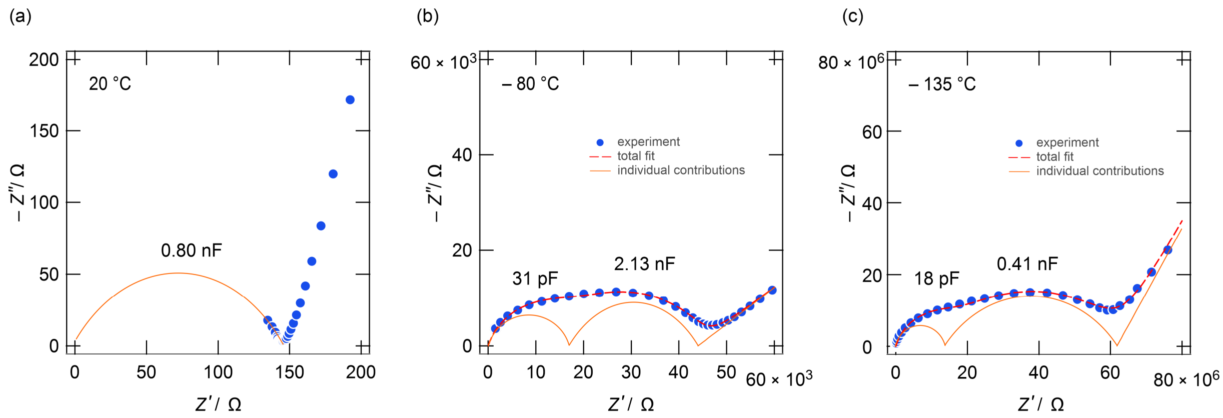

Figure S8. Nyquist representation of the complex impedance of microcrystalline $Li_{10}GeP_2S_{12}$ measured at 20 °C (a), – 80 °C (b) and – 135 °C (c) in which the imaginary part of the impedance, $-Z''$, is plotted as a function of the real part Z'. The experimental data (red) is parameterized using an equivalent circuit; the response of the individual R-CPE elements is indicated by the solid line, the response of the whole equivalent circuit by the dashed red line. The separation of the bulk and grain boundary contributions becomes more difficult as the temperature is increased. At $\vartheta = 20$ °C the bulk and grain boundary semicircles could not be resolved anymore and only a single R-CPE was used for parameterization. The associated capacitance values are also shown.

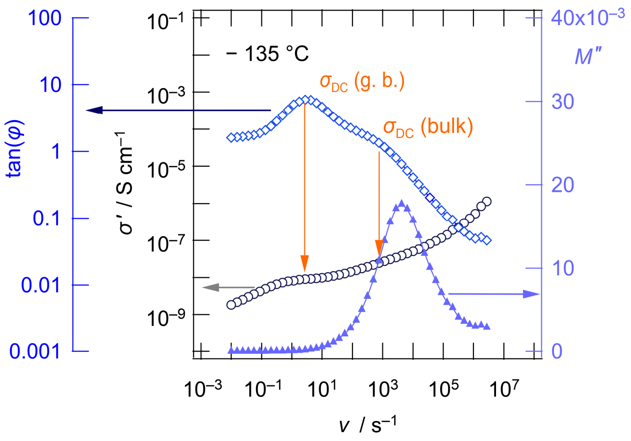

Figure S9. Conductivity spectra of $Li_{10}GeP_2S_{12}$ recorded in the frequency range from 10^{-2} to 10^{7} Hz at a temperature of – 135 °C. Additionally, the electric loss modulus M'' and the tangent of the electric loss angle $\tan(\varphi)$ are shown. The maxima (or shoulders) of the latter are used to determine the exact conductivity data points used to construct the Arrhenius plots, see Figure 6 and 8 in the main text.

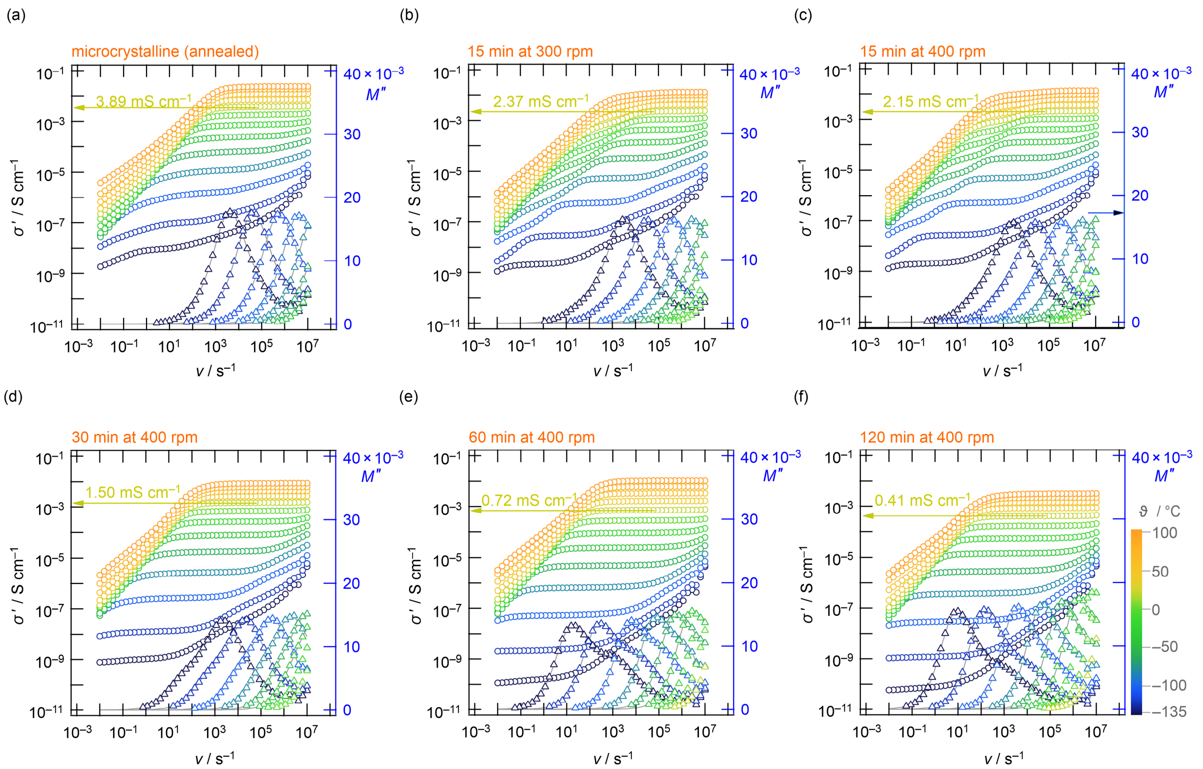

Figure S10. Conductivity spectra of $Li_{10}GeP_2S_{12}$ recorded in the frequency range 10^{-2} to 10^{7} Hz and covering a temperature range of – 135 °C to 100 °C. (a) $Li_{10}GeP_2S_{12}$ as-synthesized and annealed (microcrystalline); (b) LGPS ball milled for 15 min at 300 rpm, (c) for 15 min at 400 rpm, (d) for 30 min at 400 rpm, (e) for 60 min at 400 rpm, and (f) for 120 min at 400 rpm. A color scale of the temperature is depicted in (f). In addition to the conductivity isotherms. M'' is plotted as a function of frequency. The conductivity plateaus of the isotherms recorded at $\vartheta = 20$ °C are indicated with arrows together with the corresponding σ_{DC} values.

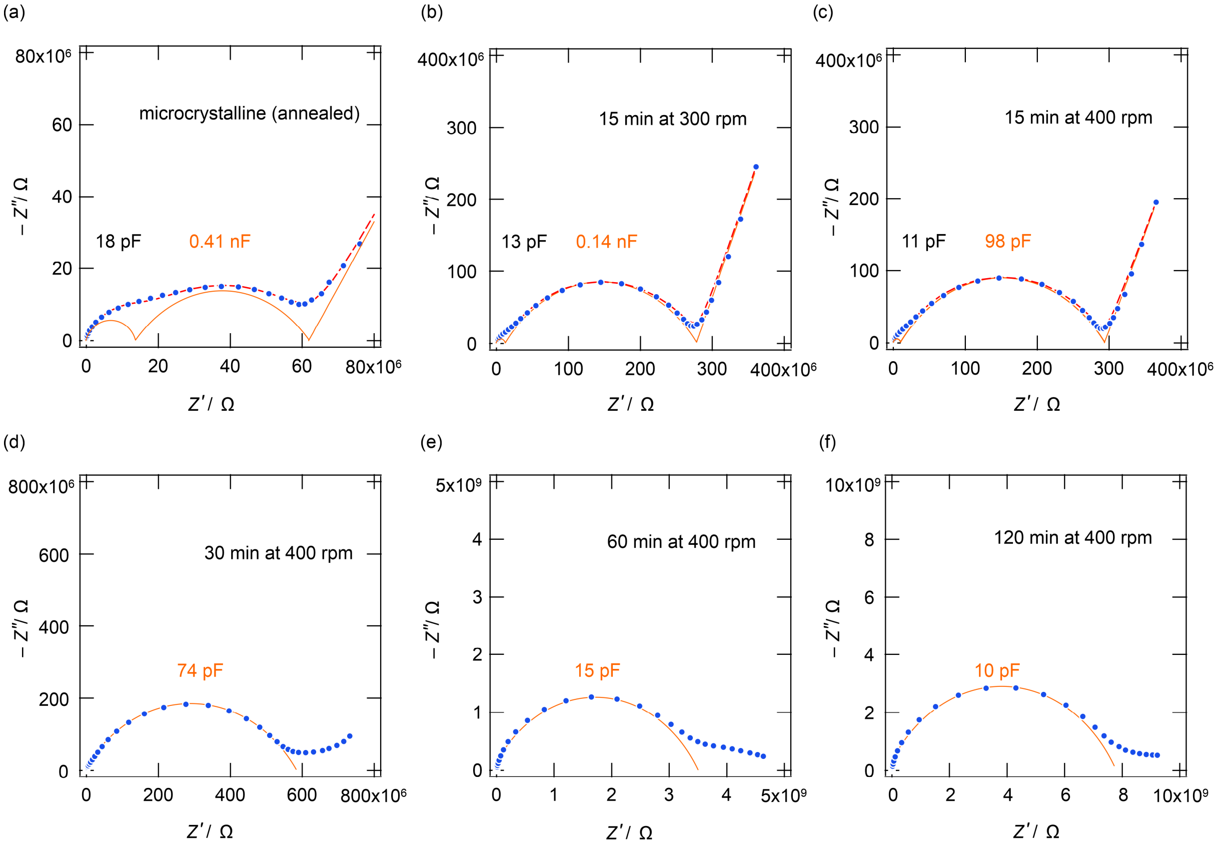

Figure S11. (a) Nyquist plots of the impedance response of $Li_{10}GeP_2S_{12}$ at ϑ = –135 °C. Data refer to the as synthesized and annealed (microcrystalline) sample, (b) data of the LGPS ball milled for 15 min at 300 rpm, (c) for 15 min at 400 rpm, (d) for 30 min at 400 rpm, (e) for 60 min at 400 rpm, and (f) for 120 min at 400 rpm. The equivalent circuits used for parameterization are included in the figures. Solid lines represent the response of the individual *R*-CPE elements. The dashed lines in (a) to (c) show the overall response.

Table S8. Results from broadband impedance analysis of the samples milled for different times and at the rotational speeds indicated

samples	$\sigma_{20°C}$ / m S cm^{-1} [a]	E_a^{bulk} / eV [a]	$E_a^{g.b.}$ / eV [b]	$E'_a{}^{g.b.}$ / eV [c]	C_{bulk} / pF cm^{-2} (138 K)	$C_{g.b.}$ / pF cm^{-2} [d] (138 K)
microcrystalline (annealed. unmilled)	3.89	0.31(1)	0.31(1)	0.26(1)	18	410
15 min at 300 rpm	2.37	0.31(1)	0.34(1)	0.24(1)	13	140
15 min at 400 rpm	2.15	0.31(1)	0.34(1)	0.25(1)	11	98
30 min at 400 rpm	1.50	0.31(2)	0.35(1)	0.25(1)	–	74
60 min at 400 rpm	0.72	0.31(1)	0.37(1)	0.35	–	15
120 min at 400 rpm	0.41	0.32(1)	0.38(1)	0.27(1)	–	10

[a] $\sigma_{20°C}$ is the dc conductivity read off from the associated (frequency independent) plateau of the conductivity isotherm.
[b] E_a^{bulk} denotes the (crystalline) bulk activation energy.
[c] the activation energy corresponding to the grain boundary (amorphous phase) responses is divided into a low temperature regime with $E_a^{g.b.}$ and a high temperature one with $E'_a{}^{g.b.}$.
[d] capacitances obtained by evaluating the electrical response with suitable equivalent circuits; values are given for grain boundary (amorphous phase) contributions $C_{g.b.}$ and for the (crystalline) bulk contributions C_{bulk} at –135 °C.

Possible Li-ion diffusion pathways

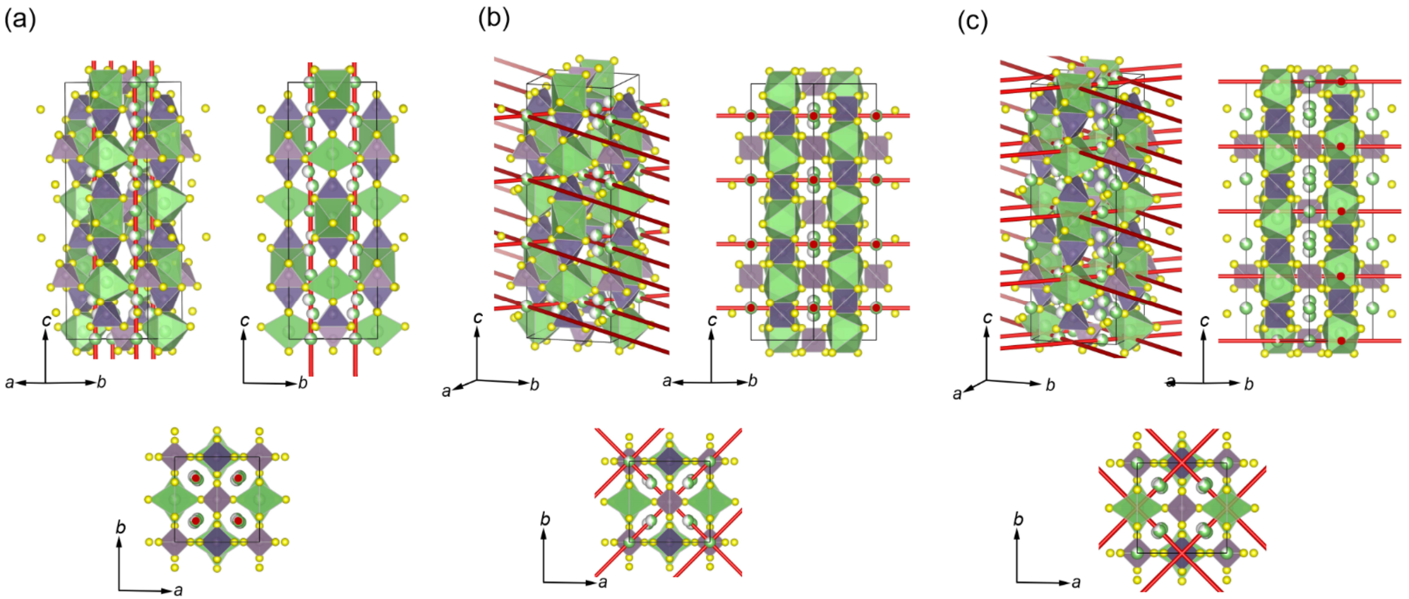

Figure S12. Possible conduction pathways along the ⟨001⟩ and ⟨110⟩ directions of LGPS. For better visualization. two-unit cells in the *c*-direction are shown. (a) [Li1S_4]-[Li1S_4]-[Li3S_4] conduction pathway along the ⟨001⟩ / *c*-direction; (b) [Li4S_6]-[Li1S_4]-[Li1S_4] conduction path along the ⟨110⟩ directions / in the *ab*-plane; (c) [Li2S_6]-[Li3S_4] conduction path along the ⟨110⟩ directions / *ab*-plane.

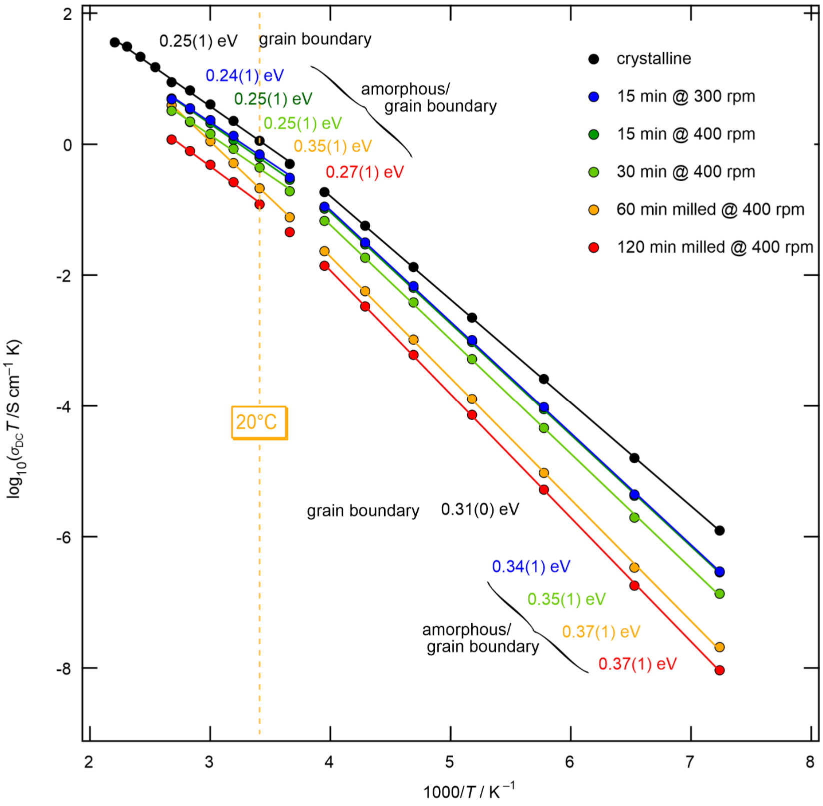

Figure S13. Arrhenius plot of the total conductivities of the various LGPS samples studies. For microcrystalline LGPS the values σ_{DC} shown here correspond to the low-frequency plateau in $\sigma'(\nu)$, which is governed by the resistive nature of the g.b. regions.

REFERENCES

(1) Kamaya. N.; Homma. K.; Yamakawa. Y.; Hirayama. M.; Kanno. R.; Yonemura. M.; Kamiyama. T.; Kato. Y.; Hama. S.; Kawamoto. K. A Lithium Superionic Conductor. *Nat. Mater.* **2011**. *10* (9). 682–686. https://doi.org/10.1038/nmat3066.

(2) Kuhn. A.; Duppel. V.; Lotsch. B. V. Tetragonal $Li_{10}GeP_2S_{12}$ and Li_7GePS_8-Exploring the Li Ion Dynamics in LGPS Li Electrolytes. *Energy Environ. Sci.* **2013**. *6* (12). 3548–3552. https://doi.org/10.1039/c3ee41728j.

(3) Hayashi. D.; Suzuki. K.; Hori. S.; Yamada. Y.; Hirayama. M.; Kanno. R. Synthesis of $Li_{10}GeP_2S_{12}$-Type Lithium Superionic Conductors under Ar Gas Flow. *J. Power Sources* **2020**. *473* (April). 228524. https://doi.org/10.1016/j.jpowsour.2020.228524.

(4) Suzuki. K.; Yageta. A.; Ikeda. Y.; Mashimo. N.; Hori. S.; Hirayama. M.; Kanno. R. Precipitation of the Lithium Superionic Conductor $Li_{10}GeP_2S_{12}$ by a Liquid-Phase Process. *Chem. Lett.* **2020**. *49* (11). 1379–1381. https://doi.org/10.1021/acs.chemmater.9b04764.

(5) Kato. Y.; Hori. S.; Saito. T.; Suzuki. K.; Hirayama. M.; Mitsui. A.; Yonemura. M.; Iba. H.; Kanno. R. High-Power All-Solid-State Batteries Using Sulfide Superionic Conductors. *Nat. Energy* **2016**. *1* (4). 1–25. https://doi.org/10.1038/nenergy.2016.30.

(6) Kuhn. A.; Gerbig. O.; Zhu. C.; Falkenberg. F.; Maier. J.; Lotsch. B. V. A New Ultrafast Superionic Li-Conductor: Ion Dynamics in $Li_{11}Si_2PS_{12}$ and Comparison with Other Tetragonal LGPS-Type Electrolytes. *Phys. Chem. Chem. Phys.* **2014**. *16* (28). 14669–14674. https://doi.org/10.1039/c4cp02046d.

(7) Bron. P.; Johansson. S.; Zick. K.; Der Günne. J. S. A.; Dehnen. S.; Roling. B. $Li_{10}SnP_2S_{12}$: An Affordable Lithium Superionic Conductor. *J. Am. Chem. Soc.* **2013**. *135* (42). 15694–15697. https://doi.org/10.1021/ja407393y.

(8) Sun. Y.; Suzuki. K.; Hara. K.; Hori. S.; Yano. T. A.; Hara. M.; Hirayama. M.; Kanno. R. Oxygen Substitution Effects in $Li_{10}GeP_2S_{12}$ Solid Electrolyte. *J. Power Sources* **2016**. *324*. 798–803. https://doi.org/10.1016/j.jpowsour.2016.05.100.

(9) Kim. K. H.; Martin. S. W. Structures and Properties of Oxygen-Substituted $Li_{10}SiP_2S_{12-x}O_x$ Solid-State Electrolytes. *Chem. Mater.* **2019**. *31* (11). 3984–3991. https://doi.org/10.1021/acs.chemmater.9b00505.

(10) Kuhn. A.; Köhler. J.; Lotsch. B. V. Single-Crystal X-Ray Structure Analysis of the Superionic Conductor $Li_{10}GeP_2S_{12}$. *Phys. Chem. Chem. Phys.* **2013**. *15* (28). 11620–11622. https://doi.org/10.1039/c3cp51985f.

(11) Patterson. A. L. The Scherrer Formula for X-Ray Particle Size Determination. *Phys. Rev.* **1939**. *56* (10). 978–982. https://doi.org/10.1103/PhysRev.56.978.

(12) Thompson. P.; Cox. D.E.;Hastings. J. B. Rietveld Refinement of Debye-Scherrer Synchrotron X-Ray Data From Al2O3. *J. Appl. Crystallopgraphy* **1987**. *20* (2). 79–83. https://doi.org/10.1007/s003300101137.

(13) Hassoun. J.; Verrelli. R.; Reale. P.; Panero. S.; Mariotto. G.; Greenbaum. S.; Scrosati. B. A Structural. Spectroscopic and Electrochemical Study of a Lithium Ion Conducting $Li_{10}GeP_2S_{12}$ Solid Electrolyte. *J. Power Sources* **2013**. *229*. 117–122. https://doi.org/10.1016/j.jpowsour.2012.11.130.

(14) Zheng. J.; Wang. P.; Liu. H.; Hu. Y. Y. Interface-Enabled Ion Conduction in $Li_{10}GeP_2S_{12}$-Poly(Ethylene Oxide) Hybrid Electrolytes. *ACS Appl. Energy Mater.* **2019**. *2* (2). 1452–1459. https://doi.org/10.1021/acsaem.8b02008.

(15) Weber. D. A.; Senyshyn. A.; Weldert. K. S.; Wenzel. S.; Zhang. W.; Kaiser. R.; Berendts. S.; Janek. J.; Zeier. W. G. Structural Insights and 3D Diffusion Pathways within the Lithium Superionic Conductor $Li_{10}GeP_2S_{12}$. *Chem. Mater.* **2016**. *28* (16). 5905–5915. https://doi.org/10.1021/acs.chemmater.6b02424.

(16) Kwon. O.; Hirayama. M.; Suzuki. K.; Kato. Y.; Saito. T.; Yonemura. M.; Kamiyama. T.; Kanno. R. Synthesis. Structure. and Conduction Mechanism of the Lithium Superionic Conductor $Li_{10+\delta}Ge_{1+\delta}P_{2-\delta}S_{12}$. *J. Mater. Chem. A* **2015**. *3* (1). 438–446. https://doi.org/10.1039/c4ta05231e.

(17) Iwasaki. R.; Hori. S.; Kanno. R.; Yajima. T.; Hirai. D.; Kato. Y.; Hiroi. Z. Weak Anisotropic Lithium-Ion Conductivity in Single Crystals of $Li_{10}GeP_2S_{12}$. *Chem. Mater.* **2019**. *31* (10). 3694–3699. https://doi.org/10.1021/acs.chemmater.9b00420.

(18) Liang. X.; Wang. L.; Jiang. Y.; Wang. J.; Luo. H.; Liu. C.; Feng. J. In-Channel and In-Plane Li Ion Diffusions in the Superionic Conductor $Li_{10}GeP_2S_{12}$ Probed by Solid-State NMR. *Chem. Mater.* **2015**. *27* (16). 5503–5510. https://doi.org/10.1021/acs.chemmater.5b01384.

(19) Heitjans. P.; Indris. S.; Wilkening. M. Solid-State Diffusion and NMR. *Diffus. Fundam.* **2005**. *2*. 45.1-45.20.
